# Genome-wide analysis of long non-coding RNAs (lncRNAs) in two contrasting rapeseed (*Brassica napus* L.) genotypes subjected to drought stress and re-watering

**DOI:** 10.1186/s12870-020-2286-9

**Published:** 2020-02-19

**Authors:** Xiaoyu Tan, Su Li, Liyong Hu, Chunlei Zhang

**Affiliations:** 1 Key Laboratory of Biology and Genetic Improvement of Oil Crops, Ministry of Agriculture and Rural Affairs, Oil Crops Research Institute of the Chinese Academy of Agricultural Sciences, Wuhan, 430062 China; 2 College of Plant Science and Technology, Huazhong Agricultural University, Wuhan, 430070 China

**Keywords:** RNA-seq, Rehydration treatments, mRNA, GO and pathway analyses, Co-expression network

## Abstract

**Background:**

Drought stress is a major abiotic factor that affects rapeseed (*Brassica napus* L.) productivity. Though previous studies indicated that long non-coding RNAs (lncRNAs) play a key role in response to drought stress, a scheme for genome-wide identification and characterization of lncRNAs’ response to drought stress is still lacking, especially in the case of *B. napus*. In order to further understand the molecular mechanism of the response of *B. napus* to drought stress, we compared changes in the transcriptome between Q2 (a drought-tolerant genotype) and Qinyou8 (a drought-sensitive genotype) responding drought stress and rehydration treatment at the seedling stage.

**Results:**

A total of 5546 down-regulated and 6997 up-regulated mRNAs were detected in Q2 compared with 7824 and 10,251 in Qinyou8, respectively; 369 down-regulated and 108 up- regulated lncRNAs were detected in Q2 compared with 449 and 257 in Qinyou8, respectively. LncRNA-mRNA interaction network analysis indicated that the co-expression network of Q2 was composed of 145 network nodes and 5175 connections, while the co-expression network of Qinyou8 was composed of 305 network nodes and 22,327 connections. We further identified 34 transcription factors (TFs) corresponding to 126 differentially expressed lncRNAs in Q2, and 45 TFs corresponding to 359 differentially expressed lncRNAs in Qinyou8. Differential expression analysis of lncRNAs indicated that up- and down-regulated mRNAs co-expressed with lncRNAs participated in different metabolic pathways and were involved in different regulatory mechanisms in the two genotypes. Notably, some lncRNAs were co-expressed with BnaC07g44670D, which are associated with plant hormone signal transduction. Additionally, some mRNAs co-located with XLOC_052298, XLOC_094954 and XLOC_012868 were mainly categorized as signal transport and defense/stress response.

**Conclusions:**

The results of this study increased our understanding of expression characterization of rapeseed lncRNAs in response to drought stress and re-watering, which would be useful to provide a reference for the further study of the function and action mechanisms of lncRNAs under drought stress and re-watering.

## Background

Drought is one of the vital factors limiting crop productivity and survival. Due to the ongoing global climate change, more and more research has focused on understanding the mechanisms of how crops resist drought stress and improve their resistance level [1–8]. Plants sense drought signals and produce second messenger substances, such as Ca^2+^, phosphatidylinositol and reactive oxygen species (ROS) [9, 10], while causing an increase in intracellular calcium ion concentration, initiating a cascade network of protein phosphorylation pathways. Finally, the target proteins are directly involved in the protection of cells, or regulate the expression of a series of specific stress-related genes through TFs (MYC/MYB, ABF, CBF/DREB, bZIP, etc.), thereby protecting the cells and improving the resistance of plants to adversity [11–13]. Although rapid developments in modern molecular biology have gradually uncovered the molecular mechanisms of plant drought resistance, developing drought-resistant plants to cope with drought stress will remain a substantive challenge in the future.

Long non-coding RNA (lncRNA) is a type of RNA transcripts which is more than 200 nucleotides in length and has no or limited protein coding abilities [14–16]. A growing body of evidence has shown that lncRNAs exert their regulatory effects on gene expression levels, involving epigenetic regulation, transcriptional regulation, and posttranscriptional regulation in the form of RNA [17–25]. With the advantage of next-generation sequencing technologies and bioinformatics approaches, many lncRNAs have been discovered in model plants, such as *Arabidopsis* [26–29], wheat [30], maize [31–33] and rice [34], indicating that lncRNAs play an important role in various biological processes of plant development and stress response. Recent research has confirmed that lncRNAs respond to abiotic stresses [31, 35, 36], including drought stress. For example, 664 drought-responsive lncRNAs were analyzed in maize [31]. Under drought stress, 2542 lncRNA candidates have been identified from *Populus trichocarpa*, 504 of which were found to be drought-responsive [37]. In *Arabidopsis*, 1832 lncRNAs changed after 2 h and/or 10 h of drought, cold, high-salt, and/or abscisic acid (ABA) treatments [29]. In maize, 664 transcripts were confirmed as drought-responsive lncRNAs, 8 out of which were proved as precursors of miRNAs [31]. In rice, pre-miRNA expression profiling indicated that miR171f is involved in the progression of rice root development and growth, as well as the response to drought stress [38]. In cotton, long intervening / intergenic noncoding RNAs (lincRNAs) XLOC 063105 and XLOC 115463, were involved in drought stress response by regulating neighboring genes [39]. Furthermore, 19 lncRNAs (17 lincRNAs and 2 natural antisense transcripts (NATs)) in foxtail millet responded to polyethylene glycol-6000 (PEG)-induced drought stress, only one of the drought-responsive lncRNA had synteny with its sorghum counterpart [40]. Qin et al. (2017) identified an *Arabidopsis* lncRNA, drought-induced lncRNA (DRIR), which responds to drought and salt stress. DRIR can be significantly activated by drought and salt stress as well as by abscisic acid (ABA) treatment [41]. In addition, in cassava, 318 lncRNAs were identified, which were responsive to cold and/or drought stress, and which are associated with hormone signal transduction, biosynthesis of secondary metabolites, and the sucrose metabolism pathway [42]. Additionally, numerous lncRNAs involved in the regulation of gene expression in response to stress have been identified and characterized in *Brassica* [43–46]. In Chinese cabbage (*Brassica rapa ssp. chinensis*), 4594 putative lncRNAs were identified to response to heat stress, 25 of which were co-expressed with 10 heat responsive genes [47]. In *Brassica rapa* L., 549 lncRNAs were identified significantly altered their expression in response to cold treatment, and short-term cold treatment induced natural antisense transcripts (NATs) in *BrFLC* and *BrMAF* genes which are involved in vernalization were identified [48]. Summanwar et al. (2019) identified 530 differentially expressed lncRNAs from the roots of clubroot-susceptible and -resistant *Brassica napus* lines. Twenty-four differentially expressed lncRNAs were identified from chromosome A08 which has been reported to confer resistance to different *P. brassicae* pathotypes [49]. In *Brassica juncea*, 1614 differentially expressed lncRNAs response to heat and drought stress, and some lncRNAs were co-expressed with TFs which are involved in abiotic stress response [50].

Rapeseed (*Brassica napus* L.) is an important oilseed crop in the world [51]. It is vulnerable to drought, which influences the production of rapeseed substantially [52–54]. Although many lncRNAs have been found in different plant species, indicating that lncRNAs can play an important role in response to abiotic stresses, a genome-wide identification and characterization of responses of lncRNAs to drought stress and rehydration treatments is still lacking, especially in *B. napus.* In order to further understand the molecular mechanisms of the response of *B. napus* to drought stress and re-watering, we compared changes in transcriptome between Q2 (a drought-tolerant genotype) and Qinyou8 (a drought-sensitive genotype) in response to drought stress and rehydration treatments at the seedling stage, and identified the lncRNAs involved in drought stress and rehydration treatments. The present study used a co-expression-based method, in which lncRNA functions were predicted, based on the functions of their co-expressed protein-coding genes [55]. Therefore, the lncRNA-mRNA co-expression network was constructed for pathway enrichment analysis. Moreover, the lncRNA-mRNA co-expression network of plant hormone signal transduction was analyzed to further explore the potential roles of differentially expressed lncRNAs in response to drought stress and re-watering.

## Results

### Phenotypes of rapeseed seedlings under drought stress (DS) and re-watering (RW) treatments

**Fig. 1:**
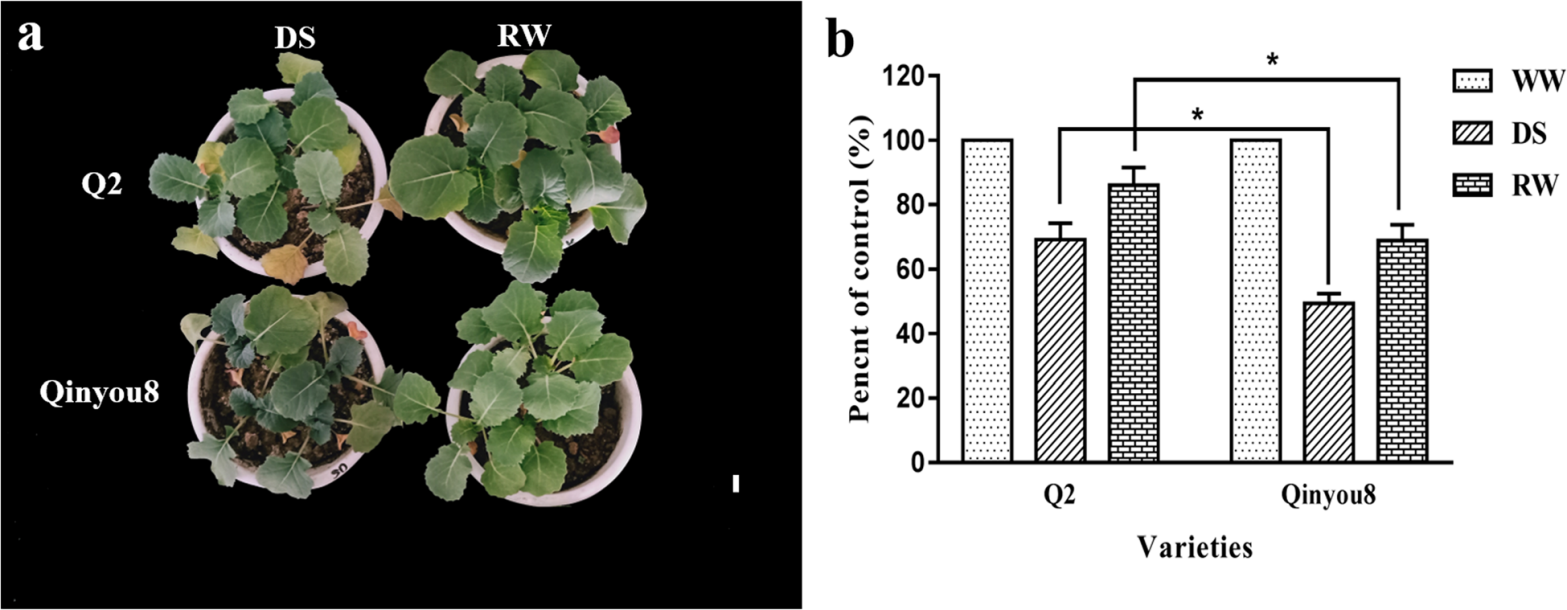
Phenotypes of seedlings under different treatments. **a** The picture show seedlings under DS and RW treatments, respectively. Bar = 1 cm. **b** Comparisons of fresh weight among the treatments. Experiments were repeated three times and vertical bars indicate standard errors. “*” indicates the significance of the difference at the 0.05 level. WW = well-watered; DS = drought stress; RW = re-watering

Rapeseed seedlings responded differently under the DS and RW treatments (Fig. 1). The fresh weight of Q2 under DS reached 70.43% of well-watered (WW), which was significantly higher than that of Qinyou8. Additionally, the fresh weight of Q2 under RW reached 82.76% of WW, which was significantly higher than Qinyou8. Therefore, we can see that the recovery ability of Q2 is better than that of Qinyou8 after re-watering.

### Differentially expressed lncRNAs and mRNAs under drought stress and re-watering

**Fig. 2:**
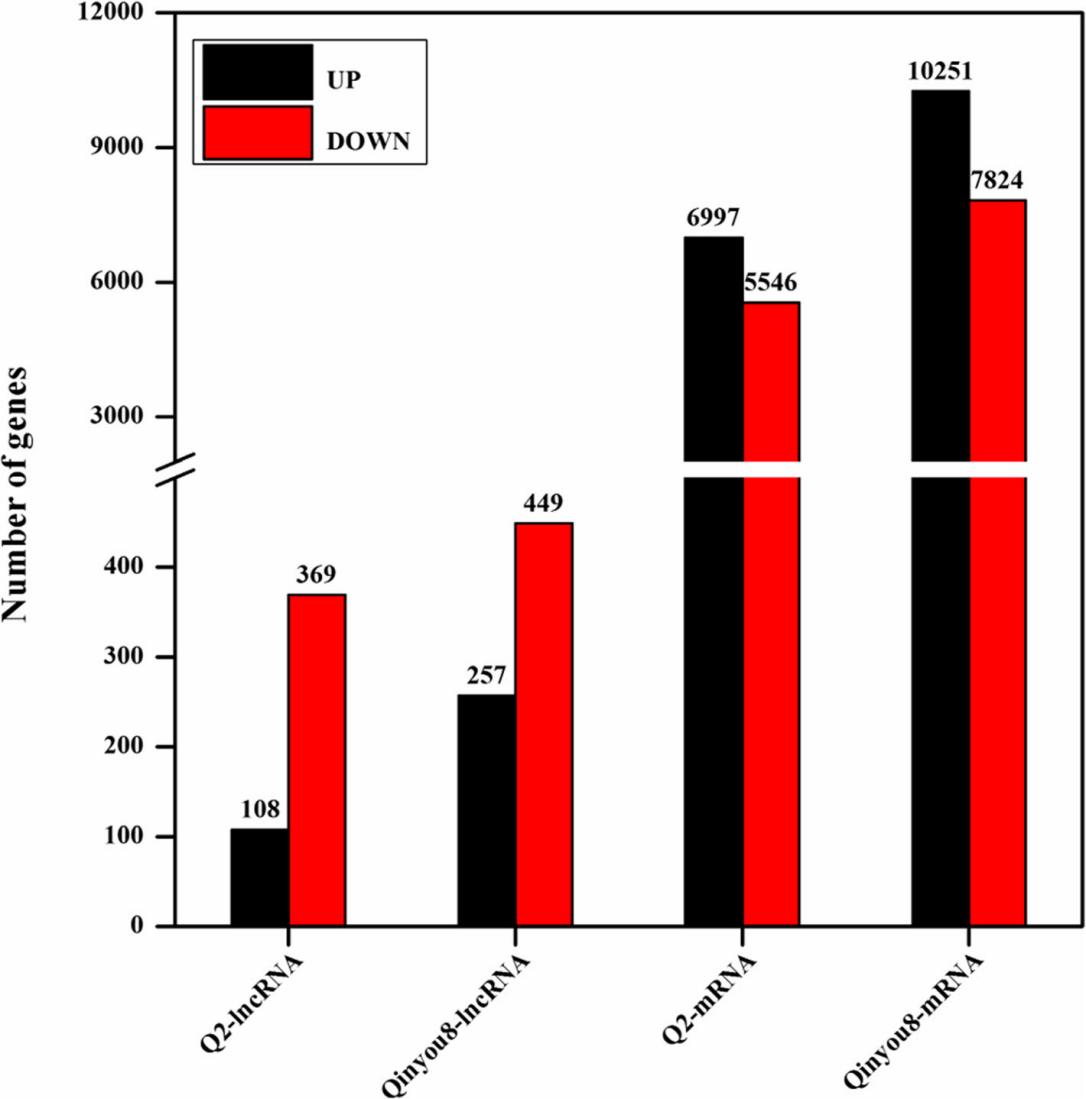
The numbers of differentially expressed lncRNAs and mRNAs in two genotypes (Q2 and Qinyou 8) in response to drought stress and re-watering treatments

In this study, RNAs were extracted from 12 samples (two treatments, two test materials, three biological replicates) and tested their quality before performing RNA sequencing (Additional file 1). We acquired clean reads by removing low-quality reads from the RNA-seq data. The QC and GC contents were calculated from clean data to assess the quality of the sequencing data (Additional file 2). The clean datasets were mapped to the *Brassica napus* L. genome. All results indicated that the RNA-seq data were very reliable. The expression level of all transcripts, including lncRNAs and mRNAs, were identified using FPKM, which was systematically estimated and the differential transcript analysis done using cuffdiff with a threshold of *q* value < 0.05. Compared with the expression of lncRNAs in drought stress, 477 lncRNAs (369 down-regulated, 108 up-regulated) of Q2 and 706 lncRNAs (449 down-regulated, 257 up-regulated) of Qinyou8 were differentially expressed after re-watering (Fig. 2). In addition, there were 12,543 mRNAs (5546 down-regulated, 6997 up-regulated) and 18,075 mRNAs (7824 down-regulated, 10,251 up-regulated) differentially expressed in Q2 and Qinyou8, respectively (Fig. 2).

### qRT-PCR validation

**Fig. 3:**
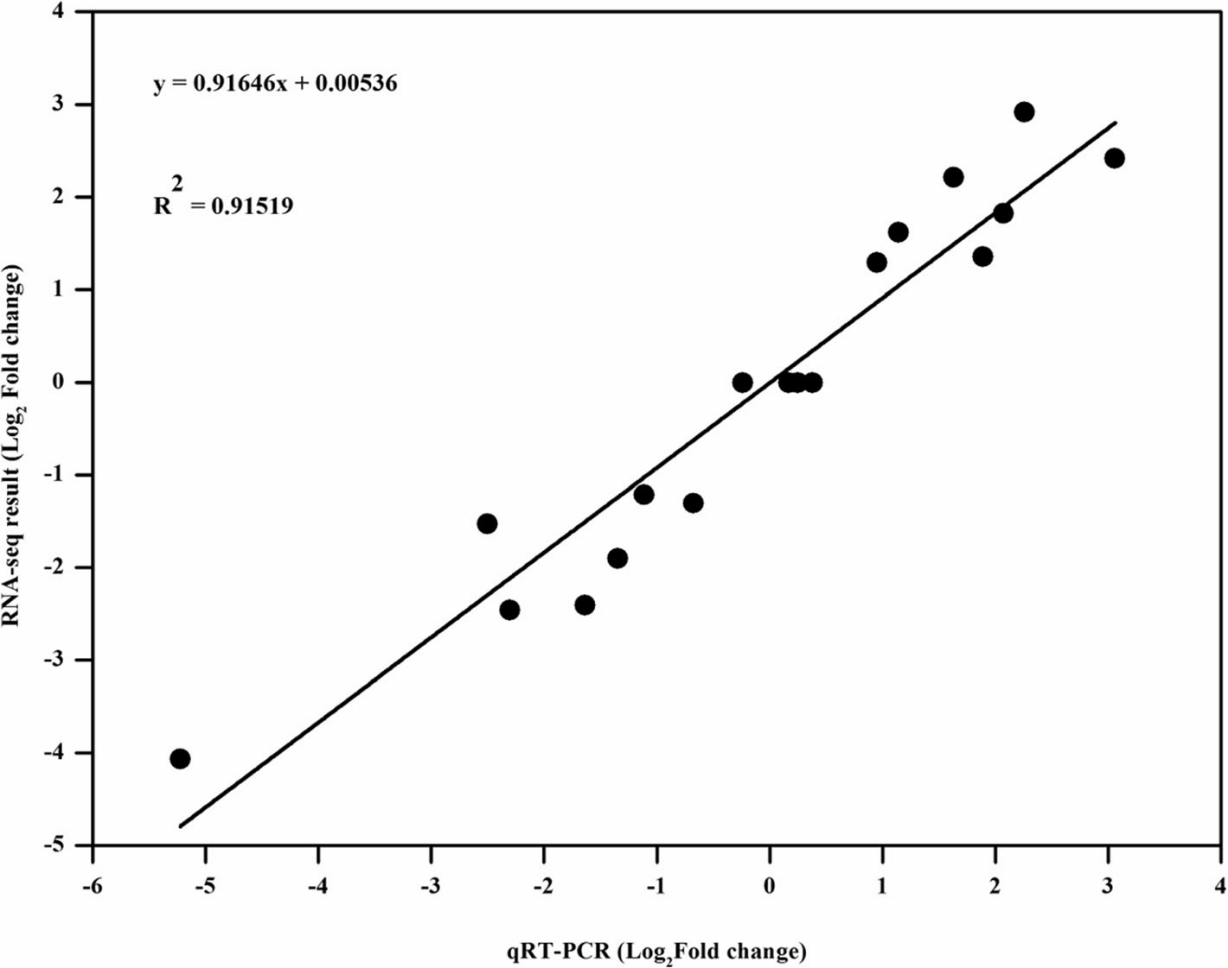
Validation of the expression levels of the lncRNAs using real-time quantitative polymerase chain reaction (RT-qPCR). The x-axis indicates the log_2_(Fold change) as measured by RT-qPCR. The y-axis indicates the log_2_(Fold change) as measured by RNA sequencing (RNA-seq). The squared of the pearson’s correlation coefficient of relative expression measured by RNA-seq and RT-qPCR was 0.91519

To validate the expression data from RNA-seq, nine lncRNAs which were differentially expressed in two genotypes were selected for real-time RT-PCR analysis. The squared of the pearson’s correlation coefficient (the determinant coefficient) of lncRNAs expression level was calculated. As shown in Fig. 3, the lncRNA expression level using RNA-seq was significantly (R^2^ = 0.91519, slope = 0.91646) correlated with those using qRT-PCR. For example, the relative expression of XLOC_012868 was increased in Q2 but decreased in Qinyou8, which was consistent with the RNA-seq result (Additional file 3). The real-time PCR results verify the expression patterns obtained with transcriptome sequencing, indicating that the lncRNAs expression profile based on RNA-seq data is reliable.

### Functions of differentially expressed lncRNAs based on lncRNA-mRNA co-expression network

To further characterize the role of differentially expressed lncRNAs, we used the lncRNA-mRNA relationship pairs to construct interactive networks. Co-expression network analysis indicated that the co-expression network of Q2 was composed of 145 network nodes and 5175 connections, while the co-expression network of Qinyou8 was composed of 305 network nodes and 22,327 connections. In Q2, there were 5175 lncRNA-mRNA pairs, including 1481 mRNAs and 145 lncRNAs, respectively (Additional file 4). Similarly, there were 22,327 lncRNA-mRNA pairs in Qinyou8, which included 3200 mRNAs and 305 lncRNAs, respectively (Additional file 4). The lncRNA-mRNA pairs with the same expression trend were much more than those with the opposite expression trend in two genotypes. There were 6 and 4 opposite-trend pairs in Q2 and Qinyou8, respectively, which suggested the candidate lncRNAs function in drought and re-watering processes (Additional file 5). It has been shown that one lncRNA may regulate multiple protein-coding genes, and vice versa [56–58]. From the co-expression network of Q2, we know that 1 mRNA may correlate with 1 to 18 lncRNAs, and that 1 lncRNA may correlate with 1 to 375 mRNAs. Moreover, the co-expression network of Qinyou8 indicated that 1 mRNA may correlate with 1 to 43 lncRNAs, and 1 lncRNA may correlate with 1 to 375 mRNAs. XLOC_071559 was the largest node in the network in both genotypes, respectively.

Studies have shown that lncRNA can indirectly affect the expression of mRNA, and can also directly bind to mRNA, thus affecting translation [59–61], shearing [62, 63], and degradation of mRNA [64]. Currently, the mechanism of interaction between lncRNA and mRNA has not yet become clear. To reveal potential functions of the differentially expressed lncRNAs under drought stress and re-watering, we analyzed Gene Ontology (GO) terms of target genes of differentially expressed lncRNAs. This analysis was performed to determine the major molecular functions, biological processes, and cellular components with which the target genes of differentially expressed lncRNAs were associated.

**Fig. 4:**
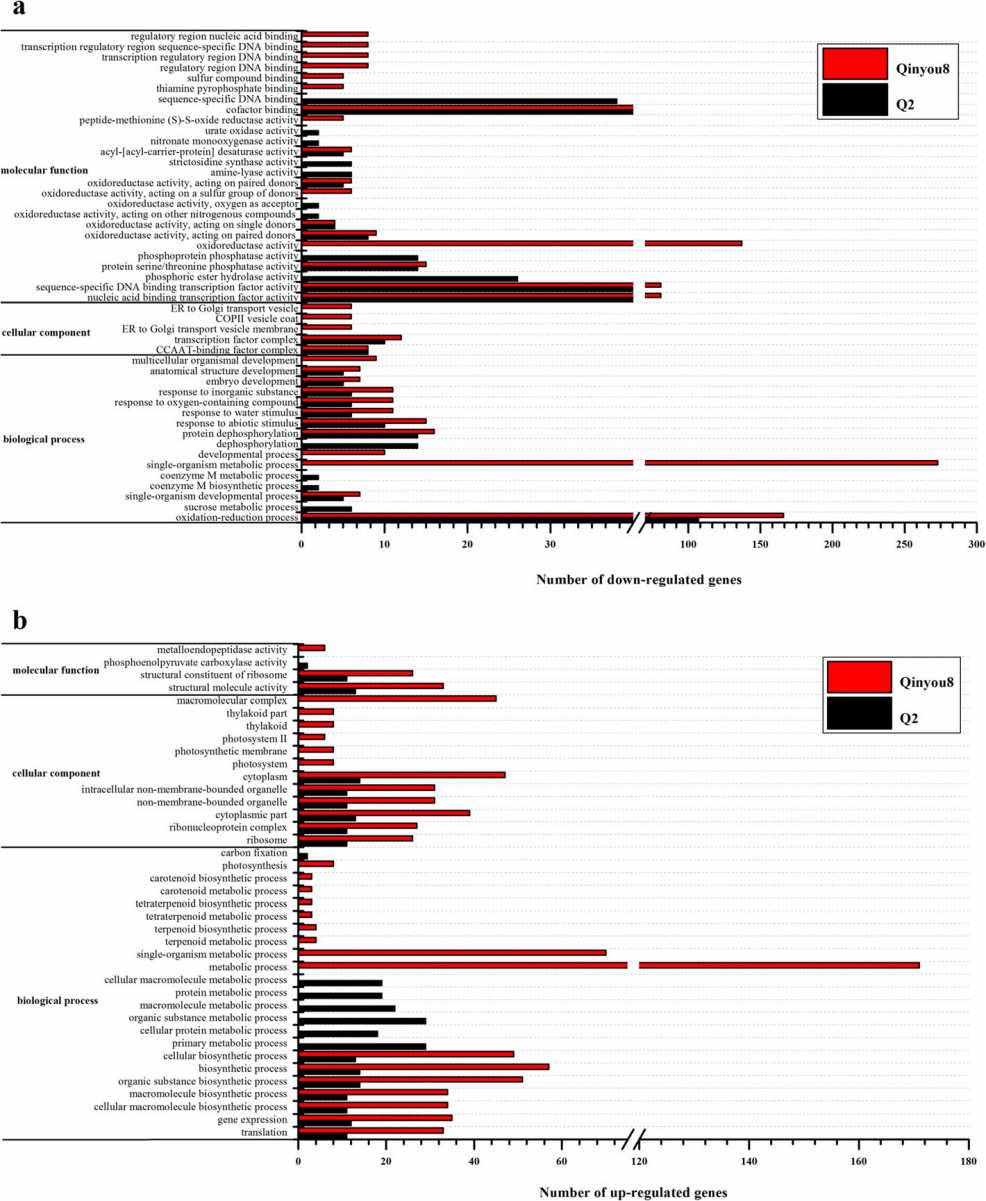
Gene Ontology (GO) classifications of the co-expressed mRNAs of the differentially expressed lncRNAs. The mRNAs co-expressed with lncRNAs are divided into three main categories by GO analysis: biological process, molecular function and cellular component. The x-axis indicates the number of genes in a sub-category, and the y-axis indicates the sub-categories. **a** The most highly enriched GO terms for the down-regulated genes in Q2 and Qinyou8. **b** The most highly enriched GO terms for the up-regulated genes in Q2 and Qinyou8

The down-regulated mRNAs, co-expressed with differentially expressed lncRNAs, were assigned to 32 and 34 significant terms in Q2 and Qinyou8, respectively (Fig. 4a). For down-regulated mRNAs co-expressed with differentially expressed lncRNAs in Q2, the most significant GO terms for biological process were oxidation-reduction process (GO:0055114), protein dephosphorylation (GO: 0006470), dephosphorylation (GO:0016311), response to abiotic stimulus (GO:0009628), response to water stimulus (GO:0009415) and sucrose metabolic process (GO:0005985). As far as molecular functions are concerned, nucleic acid binding transcription factor activity (GO:0001071), sequence-specific DNA binding transcription factor activity (GO:0003700), cofactor binding (GO:0048037), sequence-specific DNA binding (GO:0043565), phosphoric ester hydrolase activity (GO:0042578), protein serine/threonine phosphatase activity (GO:0004722) and phosphoprotein phosphatase activity (GO:0004721) were the important significantly enriched GO terms. The GO terms of transcription factor complex (GO:0005667) and CCAAT-binding factor complex (GO:0016602) were the most important significant terms for cellular components. In Qinyou8, for down-regulated mRNAs co-expressed with differentially expressed lncRNAs, the important GO terms for biological process were single-organism metabolic process (GO:0044710), oxidation-reduction process (GO:0055114), protein dephosphorylation (GO:0006470), response to abiotic stimulus (GO:0009628), response to water stimulus (GO:0009415) and protein serine/threonine phosphatase activity (GO:0004722). As far as molecular functions are concerned, three GO terms, namely, oxidoreductase activity (GO: 0016491), nucleic acid binding transcription factor activity (GO:0001071) and sequence-specific DNA binding transcription factor activity (GO:0003700), demonstrated significant enrichment. With respect to cellular components, transcription factor complex (GO:0005667) and CCAAT-binding factor complex (GO:0016602) were the most significantly enriched GO terms.

The up-regulated mRNAs co-expressed with differentially expressed lncRNAs were assigned to 23 and 31 significant GO terms in Q2 and Qinyou8, respectively (Fig. 4b). Of the enriched GO terms in the biological process category for up-regulated mRNAs co-expressed with differentially expressed lncRNAs in Q2, primary metabolic process (GO:0044238) was the most dominant group, followed by organic substance metabolic process (GO:0071704), macromolecule metabolic process (GO: 0043170), protein metabolic process (GO:0019538), cellular macromolecule metabolic process (GO: 0044260) and cellular protein metabolic process (GO:0044267). Among the molecular functions, structural molecule activity (GO:0005198) and structural constituent of ribosome (GO:0003735) were the most dominant groups in Q2. In the cellular component category, the significant terms were cytoplasm (GO:0005737), cytoplasmic part (GO:0044444), ribosome (GO:0005840), ribonucleoprotein complex (GO:0030529) and translation (GO:0006412). Additionally, in Qinyou8, the GO terms of up-regulated mRNAs co-expressed with differentially expressed lncRNAs, such as metabolic process (GO:0008152), single-organism metabolic process (GO:0044710), biosynthetic process (GO:0009058), organic substance biosynthetic process (GO:1901576), cellular biosynthetic process (GO:0044249), gene expression (GO:0010467), translation (GO:0006412) and photosynthesis (GO:0015979), were the most significantly enriched GO terms in the biological process category. With respect to molecular functions, structural molecule activity (GO:0005198) and structural constituent of ribosome (GO:0003735) were the dominant groups in Qinyou8. In the cellular component category, cytoplasm (GO:0005737), macromolecular complex (GO:0032991), cytoplasmic part (GO:0044444), non-membrane-bounded organelle (GO:0043228), ribonucleoprotein complex (GO:0030529), ribosome (GO:0005840) and photosystem II (GO: 0009523) were the dominant groups. These findings suggest that stress-responsive lncRNAs may regulate genes involved in many biological processes, including signal transduction, energy synthesis, molecule metabolism, transcription and translation, in response to drought stress and re-watering.

**Fig. 5:**
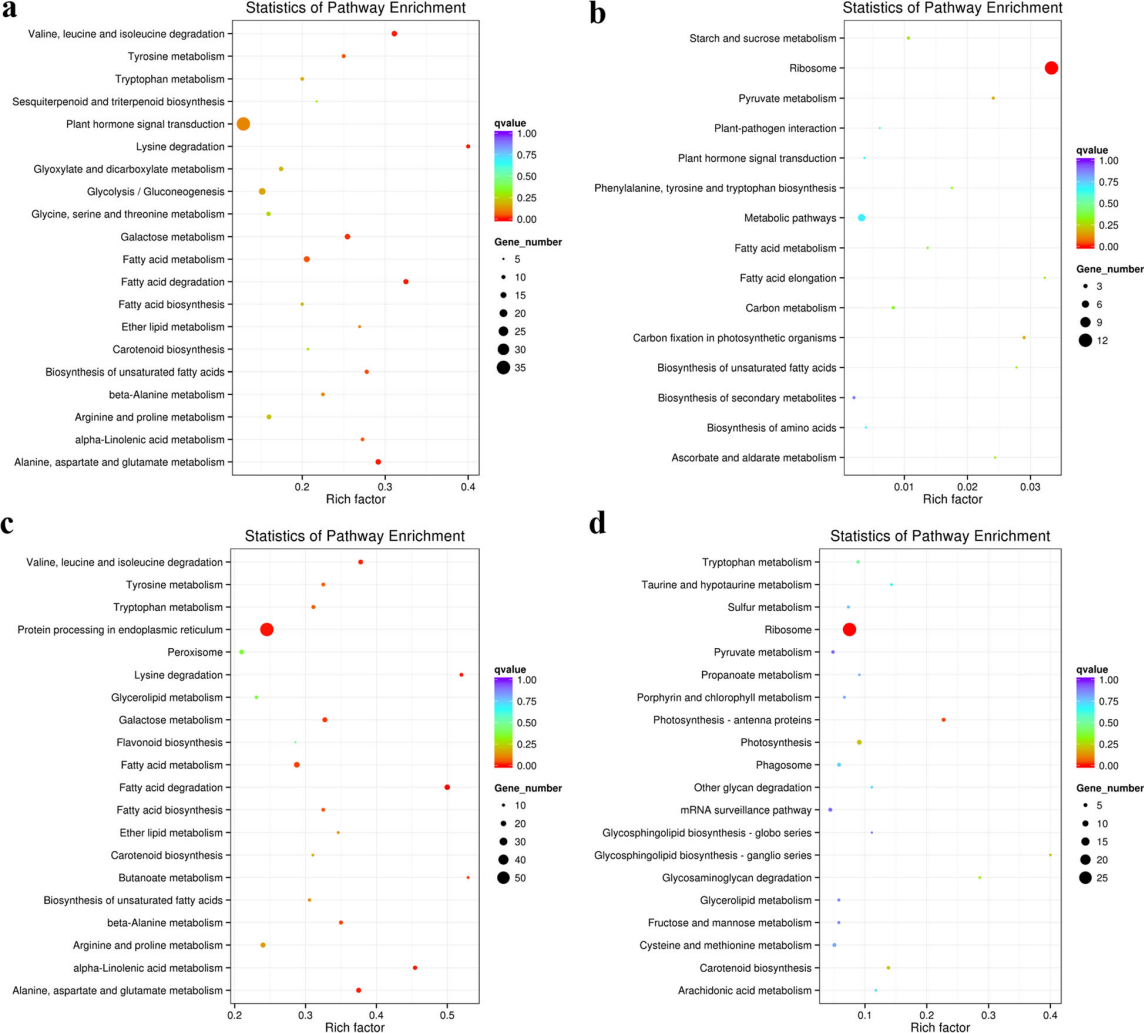
KEGG pathways analysis. Top 20 pathways for the co-expressed mRNAs of the differentially expressed lncRNAs. The y-axis corresponds to the KEGG pathway with a *q* value ≤0.05, and the x-axis shows the enrichment ratio between the number of differentially expressed genes and all UniGenes enriched in a particular pathway. The color of the dot represents q value, and the size of the dot represents the number of differentially expressed genes mapped to the reference pathways. **a** KEGG pathway classification of the down-regulated mRNAs co-expressed with lncRNAs in Q2. **b** KEGG pathway classification of the up-regulated mRNAs co-expressed with lncRNAs in Q2. **c** KEGG pathway classification of the down-regulated mRNAs co-expressed with lncRNAs in Qinyou8. **d** KEGG pathway classification of the up-regulated mRNAs co-expressed with lncRNAs in Qinyou8

We also analyzed the statistical enrichment of the mRNAs co-expressed with differentially expressed lncRNAs in KEGG. There were 18 and 18 KEGG pathways identified significantly in Q2 and Qinyou8, respectively, using pathway enrichment analysis (*p* < 0.05). KEGG analysis showed that there were 19 pathways identified that significantly related to down-regulated mRNAs co-expressed with differentially expressed lncRNAs of Q2 (Fig. 5a), including plant hormone signal transduction (ko04075), glycolysis/gluconeogenesis (ko00010), fatty acid metabolism (ko01212), valine, leucine and isoleucine degradation (ko00280), alanine, aspartate and glutamate metabolism (ko00250) and arginine and proline metabolism (ko00330). Moreover, 3 identified pathways significantly related to up-regulated mRNAs co-expressed with differentially expressed lncRNAs of Q2 (Fig. 5b), including ribosome (ko03008), carbon fixation in photosynthetic organisms (ko00710), and pyruvate metabolism (ko00620). In Qinyou8, 17 pathways were identified that were significantly related to down-regulated mRNAs co-expressed with differentially expressed lncRNAs and 7 identified pathways significantly related to up-regulated mRNAs co-expressed with differentially expressed lncRNAs. The most down-regulated mRNAs co-expressed with lncRNAs of Qinyou8 were significantly enriched for protein processing in endoplasmic reticulum (ko04141), fatty acid metabolism (ko01212), fatty acid degradation (ko00071), alanine, aspartate and glutamate metabolism (ko00250), galactose metabolism (ko00052), as well as arginine and proline metabolism (ko00330) (Fig. 5c), while the most up-regulated mRNAs co-expressed with lncRNAs of Qinyou8 denoted their involvement in ribosome (ko03010), photosynthesis (ko00195), and photosynthesis - antenna proteins (ko00196) (Fig. 5d).

### Identification of TFs under drought stress and re-watering

**Fig. 6:**
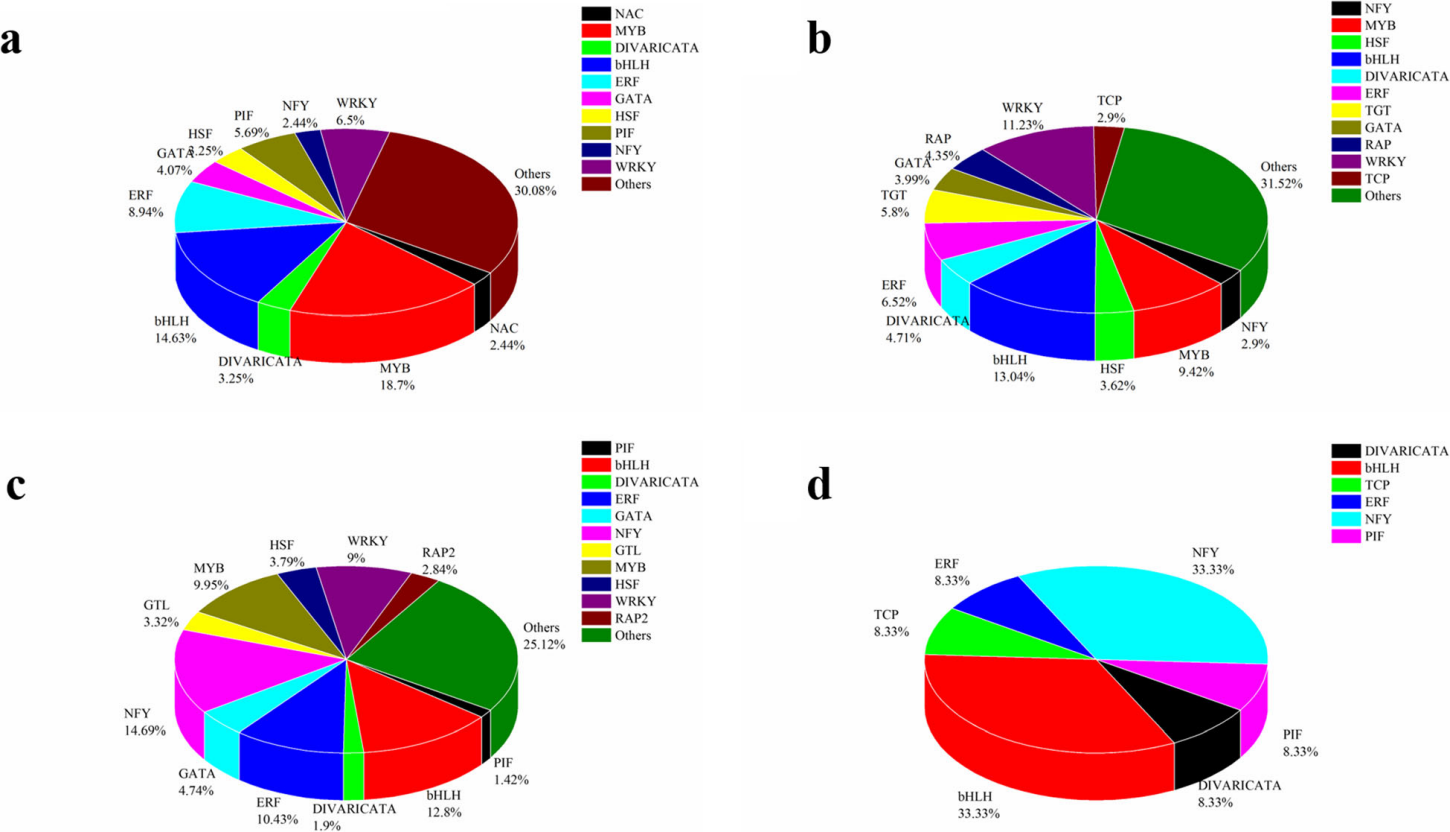
Distribution of transcription factor gene families in two genotypes under drought stress and rehydration conditions. **a** Distribution of transcription factors specially expressed in Q2. **b** Distribution of transcription factors specially expressed in Qinyou8. **c** Distribution of transcription factors expressed in both genotypes. **d** Distribution of transcription factors conversely expressed in the two genotypes

Under drought stress, TFs can be used as regulators of drought stress, and they would bind to cis-acting elements in the promoter region of related genes to regulate the expression of downstream genes [65]. In our research we found that there were 334 differentially expressed genes in Q2, and 487 differentially expressed genes in Qinyou8; when compared to the TF database of *Arabidopsis*, 211 TFs were found to be co-expressed in two genotypes, and 12 TFs were conversely expressed in two genotypes. In addition, 123 TFs that were specifically expressed in Q2 were classified into 38 groups; 10 of these TF families comprised 69.92% of these groups, including MYB (23 TFs), basic helix-loop-helix (bHLH) (18 TFs), ERF (11 TFs), WRKY (8 TFs), PIF (7 TFs), GATA (5 TFs), DIVARICATA (4 TFs), HSF (4 TFs), NAC (3 TFs) and NFY (3 TFs) (Fig. 6a). Moreover, 276 TFs, classified into 56 TF groups were specifically expressed in Qinyou8, in which 11 TF families accounted for 68.48% of 276 TFs; these included MYB (26 TFs), basic helix-loop-helix (bHLH) (36 TFs), ERF (18 TFs), WRKY (31 TFs), GATA (16 TFs), DIVARICATA (13 TFs), HSF (10 TFs), RAP (11 TFs), TCP (12 TFs), NFY (8 TFs) and TGT (8 TFs) (Fig. 6b). Thirty-nine groups of TFs were co-expressed in two genotypes, including NFY (31 TFs), bHLH (27 TFs), WRKY (19 TFs), MYB (21 TFs), ERF (22 TFs), and GATA (10 TFs) (Fig. 6c). Additionally, 6 groups conversely expressed between two genotypes (con-expression TFs); these included bHLH (4 TFs), NFY (4 TFs), DIVARICATA (1 TFs), ERF (1 TFs), PIF (1 TFs) and TCP (1 TFs) (Fig. 6d). We also analyzed the relationship between the differentially expressed lncRNAs and transcription factors. The sequence information of TFs was obtained and used for co-expression analysis with lncRNAs. In Q2, 57 TFs belonging to the 20 TF families were found co-expressed with 57 differentially expressed lncRNAs (Additional file 6), while there were 94 TFs belonging to the 24 TF families were found co-expressed with 172 differentially expressed lncRNAs in Qinyou8 (Additional file 6).

## Discussion

Several recent studies have revealed that lncRNAs play an important role in response to drought stress [31, 39, 41, 66]. Accordingly, we constructed lncRNA and mRNA libraries, and annotated, identified, and verified those lncRNAs that are involved in drought stress and re-watering.

### Differential mRNAs and lncRNAs expression in two contrasting genotypes under drought stress and re-watering

**Fig. 7:**
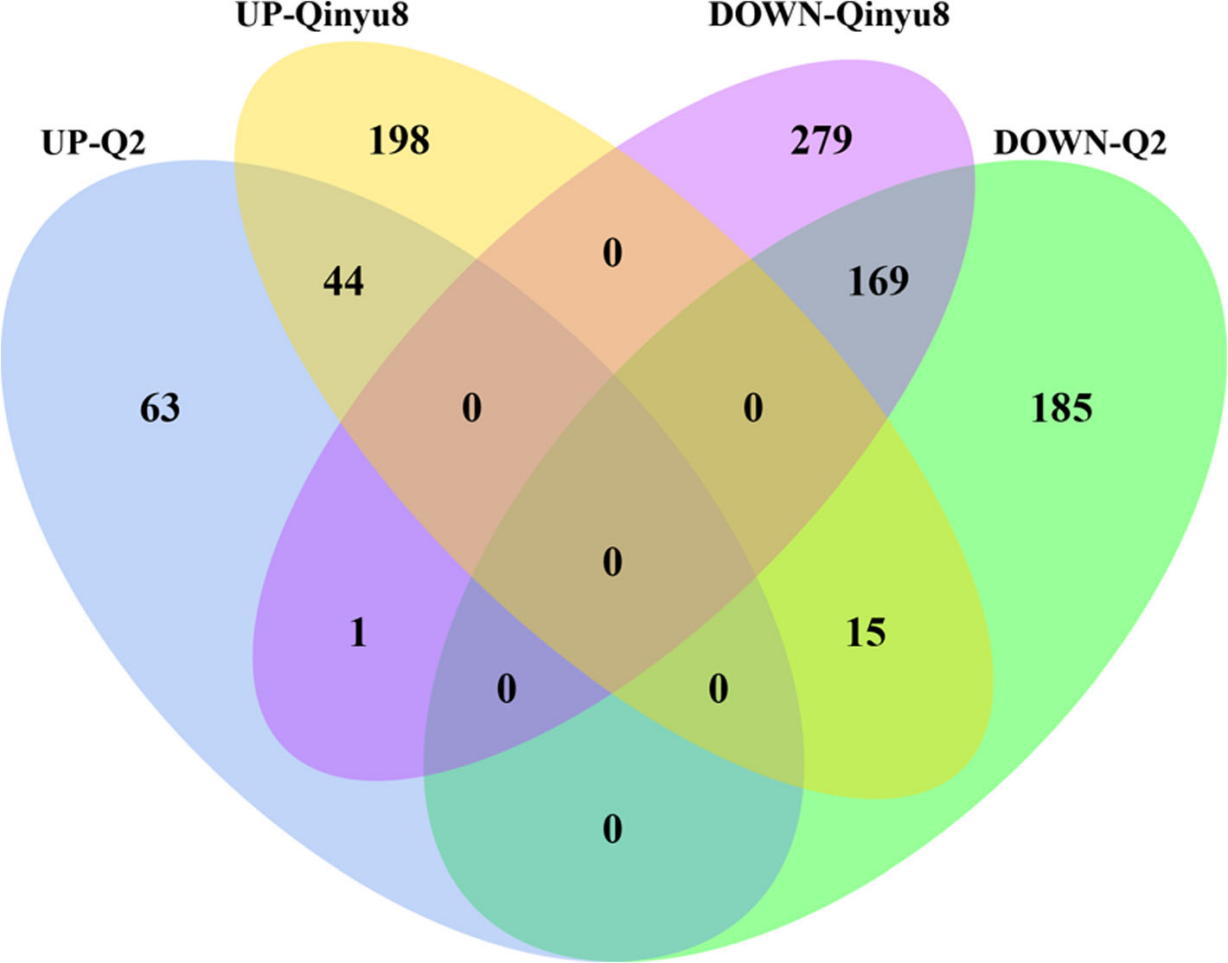
Venn diagram showing the number of unique and common differentially expressed lncRNAs in both genotypes under drought stress and rehydration conditions

In our research, we systematically identified and analyzed *B. napus* mRNAs and lncRNAs, which respond to drought stress and rehydration. In the comparison groups with two different genotypes, 5546 down-regulated and 6997 up-regulated mRNAs were detected in Q2 compared to 7824 and 10,251 in Qinyou8, respectively; 369 down-regulated and 108 up-regulated lncRNAs were detected in Q2 compared to 449 and 257 in Qinyou8, respectively. Interestingly, we found that there were 229 lncRNAs (169 down-regulated, 44 up-regulated) in both genotypes, among which, 1 lncRNA XLOC_012868 was up-regulated in the drought-tolerant genotype and down-regulated in drought-susceptible genotype; conversely, 15 lncRNAs were down-regulated in the drought-tolerant genotype and up-regulated in the drought-susceptible genotype (Fig. 7). From the above, we know that the response of these two genotypes is different under drought stress and rehydration conditions. In Qinyou8, the number of differentially expressed mRNAs and lncRNAs was significantly higher than Q2.

Altered splicing is one of the mechanisms for lncRNA transcripts to affect gene expression in many physiological processes [67–69]. In Q2, 477 lncRNA transcripts from 469 lncRNA genes were identified, in which 8 lncRNA coding genes were alternatively spliced. Similarly, in Qinyou8, 706 lncRNA transcripts from 688 lncRNA genes were identified, in which 18 lncRNA coding genes were alternatively spliced (Additional file 7). These alternately spliced lncRNA coding genes may be involved in drought and re-watering processes. Additionally, 9 identified lncRNAs were chosen for qRT-PCR validation, and the results confirmed the sequencing results.

### Differentially expressed lncRNAs specifically enriched in GO and KEGG pathways

With advances in next-generation sequencing technology, many investigations have shown that lncRNAs exert their regulatory effects on gene expression levels, including epigenetic regulation, transcriptional regulation, and post-transcriptional regulation in the form of RNA [19]. It is known that sequence-specific DNA binding transcription factor activity [42, 70], response to stimulus [71], response to abiotic stimulus [70], biosynthetic process [70], structural constituent of ribosome [58], photosynthesis [72] and oxidoreductase activity [72], which are regulated by some lncRNAs, have been reported in response to abiotic stresses, and these GO terms were identified in this study. To determine the similarity and differences between the two genotypes, the significantly enriched GO terms were compared. In our study, there were more significant GO terms in Qinyou8 than Q2 under drought stress and re-watering, indicating that there were differences in responses to drought stress and re-watering between the two genotypes. We found that phosphoprotein phosphatase activity, protein metabolic process, and sequence-specific DNA binding were significantly and specially enriched in Q2, while single-organism metabolic process, photosynthesis, and oxidoreductase activity were significantly and specially enriched in Qinyou8. Additionally, lncRNAs have been recognized as powerful regulators of pathways in response to drought stress, including ribosome, photosynthesis [73], and plant hormone signal transduction [42, 74]. The ribosome pathway was simultaneously significant in both genotypes, and the differential lncRNA target genes were up-regulated in this pathway. It is worth noting that the pathway of plant hormone signal transduction was significantly and specially enriched in Q2, a total of 36 mRNAs co-expressed with 41 lncRNAs were assigned to plant hormone signal transduction. Furthermore, many down-regulated mRNAs co-expressed with lncRNAs involved in protein processing in the endoplasmic reticulum, and up-regulated mRNAs co-expressed with lncRNAs belonging to photosynthesis were significantly and specially enriched in Qinyou8. A total of 7 and 5 mRNAs co-expressed with lncRNAs were assigned into photosynthesis and photosynthesis-antenna proteins, respectively. The genes involved in photosynthesis were generally down-regulated by drought [75, 76]. Compared with the DS treatment, photosynthesis (ko00195) and photosynthetic antenna protein (ko00196) pathways were significantly enriched in Qinyou8 under the RW treatment, indicating that the short-term drought stress did not cause significantly damage to photosynthesis of Q2, but did some damage to Qinyou8. In Qinyou8, the genes involved in photosynthesis (ko00195) and photosynthetic antenna protein (ko00196) were up-regulated to restore normal photosynthesis and thus restore growth. These results indicate that lncRNAs could play a role in many biological processes responding to drought stress and re-watering through regulating gene network, and that up- and down-regulated mRNAs co-expressed with lncRNAs participate in different metabolic pathways and are involved in different regulation mechanisms. Taken together, our results suggest that the two different genotypes implement divergent mechanisms to modulate the response to drought stress and re-watering treatment.

### Analysis of plant signal transduction using lncRNA-mRNA co-expression network

**Fig. 8:**
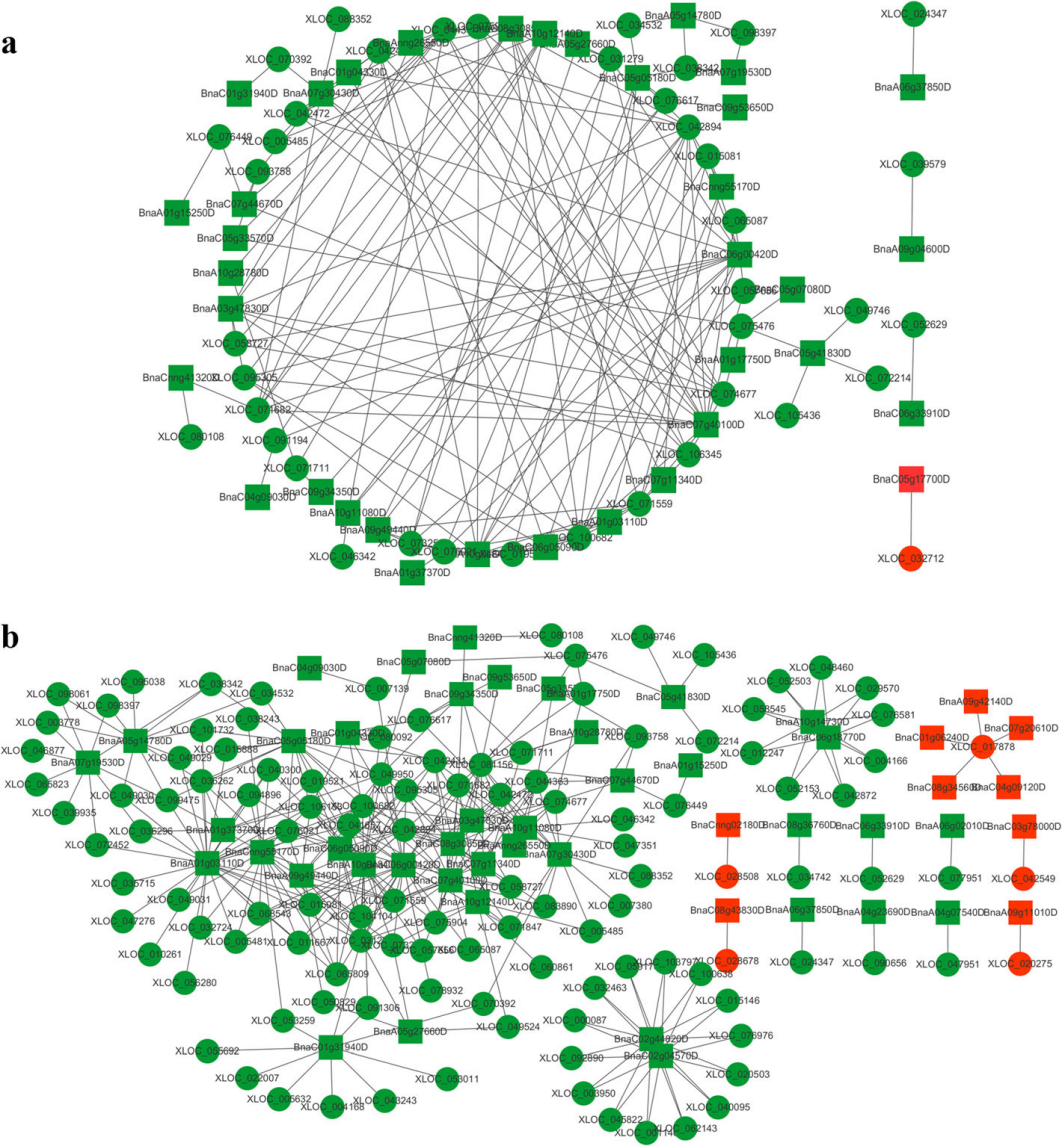
LncRNA-mRNA-network analysis. The circle and rectangle nodes represent lncRNAs and protein-coding genes, respectively. The up-regulated and down-regulated nodes are separately colored in red and green. Edges show regulatory interactions among nodes. **a** 40 lncRNAs interacted with 36 mRNAs in the meaningful “plant hormone signal transduction” in Q2. **b** 120 lncRNAs interacted with 51 mRNAs in the meaningful “plant hormone signal transduction” pathway in Qinyou8

Regulation on the co-expression network may be the possible mechanisms in response to stress for lncRNAs [18, 31]. Although a large number of lncRNAs were identified to be related with many biological processes, a limited number of lncRNAs were screened out to contribute to plant hormone signal transduction by using lncRNA-mRNA co-expression analysis. In Q2, the co-expression network of plant hormone signal transduction contained 157 matched lncRNA-mRNA pairs, including 41 lncRNAs and 36 mRNAs (Fig. 8a and Additional file 8). The co-expression network of plant hormone signal transduction of Qinyou8 was composed of 120 lncRNAs and 51 mRNAs with 352 matched lncRNA-mRNA pairs (Fig. 8b and Additional file 8). The lncRNAs involved in plant hormone signal transduction had the same expression direction with the target genes in two genotypes, proving the expression of lncRNAs promoted the function of the target genes. In this pathway, target genes of differentially expressed lncRNAs were involved in auxin, cytokinin, gibberellin, and abscisic acid signaling pathways in both genotypes. Some target genes of differentially expressed lncRNAs related to the ethylene and salicylic acid signaling pathways were specifically expressed in Q2, while target genes of differentially expressed lncRNAs involved in the two signaling pathways of BR and jasmonic acid were specifically expressed in Qinyou8. Among these signaling pathways, more of the mRNAs, which co-expressed with differentially expressed lncRNAs, were associated with the ABA signaling pathways than those of other phytohormones, which is consistent with previous studies that had considered ABA to be an early warning signal for plant responses to drought stress [77, 78].

**Auxin (IAA)** as a phytohormone, is essential for signaling, transport, growth and development of a plant [79]. Auxin binds to the TRANSPORT INHIBITOR RESPONSE 1/AUXIN SIGNALLING F-BOX proteins (TIR1/AFBs) and the AUXIN/INDOLE-3-ACETIC ACID (Aux/IAA) proteins. When the level of IAA is low, the Aux/IAA protein forms a heterodimer with the auxin response factor (ARF) to inhibit gene transcription. Conversely, the Aux/IAA protein is degraded, which results in derepression of the ARF transcriptional regulation and expression of the auxin response gene [80]. Currently, IAA early response genes mainly include AUXIN/INDOLE-3-ACETIC ACID (Aux/IAA), Gretchen Hagen 3 (GH3) and Small Auxin-Up RNAs (SAUR), which are auxin-induced primitive expression genes [81]. Among them, Aux/IAA protein plays a very important role in the IAA signal transduction pathway, and it acts as a transcriptional repressor in the signal transduction pathway [82]. The GH3 gene encodes an auxin-binding enzyme that acts as a feedback regulator of auxin by reducing the level of beneficial auxin [83]. In Q2, the co-expression network of IAA signal transduction contained 21 matched lncRNA- mRNA pairs, including 16 lncRNAs and 3 mRNAs. In Qinyou8, the co-expression network of IAA signal transduction contained 56 matched lncRNA- mRNA pairs, which included 46 lncRNAs and 12 mRNAs. Drought stress and re-watering regulated the expression of *Aux/IAA* (1 differentially expressed mRNA co-expressed with lncRNAs in Q2, and 6 differentially expressed mRNAs co-expressed with lncRNAs in Qinyou8), and *GH3* (1 differentially expressed mRNA co-expressed with lncRNAs in Q2, and 3 differentially expressed mRNAs co-expressed with lncRNAs in Qinyou8) genes in the two genotypes. In Q2, down- regulated XLOC_042431, XLOC_071559, XLOC_095305, XLOC_100682, XLOC_019521 and XLOC_042894, targeting down-regulated BnaC06g05090D (encoding *Aux/IAA*), possibly take part in regulating the IAA signal transduction pathway in a positive way. Furthermore, down-regulated XLOC_098397, XLOC_034532 and XLOC_038342, targeting down-regulated BnaA05g14780D (encoding *GH3*), facilitating the level of beneficial auxin. It is suggested that down regulation of these lncRNAs expression in Q2 led to enhance IAA signal, which may accelerate vegetative growth by cell enlargement. In Qinyou8, up-regulated XLOC_017878, XLOC_042549, and XLOC_028678, targeting up-regulated BnaC01g06240D, BnaC03g78000D, and BnaC08g43830D (encoding *Aux/IAA*), respectively. Additionally, up-regulated XLOC_017878, targeting up-regulated BnaA09g42140D and BnaC08g34560D (encoding *GH3*). Up regulation of these lncRNAs expression in Qinyou8 led to weakened IAA signal, which may inhibit vegetative growth.

Cytokinin (CK) plays an important role in various physiological functions in plants, such as promoting cell division, inducing shoot formation and promoting its growth [79]. Cytokinin signaling is based on a two-component signaling system (TCS), which is mainly composed of *Arabidopsis* histidine kinases (AHKs), *Arabidopsis* histidine phosphotransfer proteins (AHPs) and *Arabidopsis* response regulators (ARRs). Firstly, the cytokinin receptor binds to cytokinin and then to autophosphorylates. Subsequently, it transfers the phosphate group to a phosphotransferase of the cytoplasm through transmembrane transport; the phosphorylated AHPs can then enter the nucleus and transfer the phosphate group to the response regulator, thereby inducing gene expression and regulating plant growth and development [84]. The type-B response regulators (B-ARR) function as positive regulators of cytokinin signaling, while the type-A response regulators (A-ARR) function as a downstream signal that acts as the negative regulators of cytokinin signaling and also inhibits the signal transmission of B-ARR [85]. In Q2, the co-expression network of CK signal transduction contained 7 matched lncRNA-mRNA pairs, including 7 lncRNAs and 3 mRNAs. Down-regulated BnaA01g17750D is involved in encoding *B-ARR* gene, was targeted by down-regulated XLOC_075476 and XLOC_074677, indicating that down-regulated XLOC_075476 and XLOC_074677 are likely to weakened CK signal, which may inhibit the seedling growth of Q2. In Qinyou8, the co-expression network of IAA signal transduction contained 27 matched lncRNA- mRNA pairs, which included 25 lncRNAs and 5 mRNAs. Down-regulated BnaC06g18770D is involved in encoding *A-ARR* gene, was targeted by 9 down-regulated lncRNAs. It is suggested that 9 down-regulated lncRNAs of Qinyou8 are likely to enhance CK signal, which may benefit rapeseed seedling growth.

Gibberellin (GA) plays an important role in all stages of plant growth and development, and it participates in various physiological processes that regulate plant growth and development. One of the most significant effects is the promotion of internode elongation, which promotes plant growth [86]. *GIBBERELLIN INSENSITIVE DWARF1* (*GID1*) receptor is a soluble protein that is localized to both cytoplasm and nucleus. GID1 protein can specifically bind to active GA and further bind with DELLA protein to form GID1-GA-DELLA [87]. By mediating the degradation of or inhibiting the activity of DELLA protein, the GID1-GA-DELLA disinhibits DELLA protein from the GA reactive system, and then activates the GA reactive gene [88]. When GA is at a low level, GID1 does not bind to GA, allowing the DELLA protein to bind to the gibberellin responsive gene and inhibit its activity, thereby inhibiting plant growth. When GA is at a high level, GID1 can sense the GA signal, forming GID1-GA-DELLA to degrade DELLA protein, which inhibits the repressing of DELLA on GA signaling [89]. In Q2, the co-expression network of GA signal transduction contained 8 matched lncRNA-mRNA pairs, including 8 lncRNAs and 4 mRNAs. In Qinyou8, the co-expression network of GA signal transduction contained 31 matched lncRNA-mRNA pairs, which included 30 lncRNAs and 4 mRNAs. Two mRNAs (BnaA07g19530D and BnaCnng55170D) co-expressed with lncRNAs, which were down-regulated in both genotypes and which respond to drought stress and re-watering, were annotated to *GID1*. Down-regulated *GID1* genes prevented the formation of complexes with GA and DELLA proteins, resulting in the binding of the DELLA protein to the gibberellin response gene, thereby inhibiting seedling growth.

Abscisic acid (ABA) as a signal molecule for plants to perceive stress [90], plays an important role in preventing plant water loss, regulating stomatal opening, and maintaining the balance of cell permeability [90]. ABA binds its receptor PYR/PYL/RCAR (pyrabactin resistant/PYR-like/regulatory component of ABA) and inhibits the activity of PP2C (protein phosphatases type-2C), which leads to the autophosphorylation of downstream SnRK2 (sucrose non-fermenting 1-related subfamily 2 kinases) and the phosphorylation of downstream ABF transcription factors, regulating the expression of stress-related genes [91, 92]. BnaC07g44670D is homologous to gene ABF (AT4G34000) in *Arabidopsis thaliana*, which has been reported to be an important gene involved in ABA signaling [93]. In Q2, the co-expression network of ABA signal transduction contained 119 matched lncRNA-mRNA pairs, including 37 lncRNAs and 24 mRNAs. In Qinyou8, the co-expression network of ABA signal transduction contained 207 matched lncRNA-mRNA pairs, which included 73 lncRNAs and 25 mRNAs. In our research, we identified that lncRNAs that co-expressed with BnaC07g44670D, differed between the two genotypes. XLOC_074677, XLOC_093758, XLOC_044363 and XLOC_076449, which co-expressed with BnaC07g44670D, were down- regulated in the two genotypes. XLOC_081156 which co-expressed with BnaC07g44670D, was only down-regulated in Qinyou8. These findings suggest that altered lncRNAs may be involved in “plant hormone signal transduction” and regulated differently in the two genotypes. The up-regulation of ABF in response to drought stress can trigger stomatal closure and seed dormancy [94]. The down-regulation of ABF in response to re-watering led to weakened ABA signal, which may alleviate rapeseed seedling growth inhibition by ABA.

### Transcription factors functioned under drought stress and re-watering

Transcription factors have been confirmed to play a crucial role in regulating drought stress in plants [89, 95, 96]. Previously, MYB [97, 98], bHLH [99, 100], WRKY [101], ERF [102], NFY [103], GATA [104], PIF [105, 106], ABA-INDUCIBLE BHLH-TYPE TRANSCRIPTION FACTOR (AIB) [107], HSF [108], and bZIP [109] had been proposed as being responsive to abiotic stresses. In this study, these TFs were induced to express under drought stress and re-watering.

Studies have shown that MYB was involved in response to abiotic stress, which could be induced by ABA, to participate in the regulation of waxy synthesis pathway of drought stress response [110], and that it promoted the drought resistance of plants by promoting stomatal closure and reducing leaf water loss [111, 112]. At present, the research on the possible role of bHLH TFs in plant response to drought stress mainly focuses on stomatal development, trichome development, root hair development, and abscisic acid (ABA) sensitivity [99]. The bHLH-type transcription factor AtAIB depended on ABA signal transduction pathway to participate in the drought resistance response in *Arabidopsis* [113]. It was found that overexpression of *OsbHLH148* in rice induced up-regulation of *OsDREB*, *OsJAZ* and other related genes involved in stress response, and in the jasmonic acid signaling pathway, indicating that *OsbHLH148* regulated the expression of jasmonic acid signaling pathway-related genes as a starting response factor during drought stress [114]. Among expressed TFs, the most specifically expressed in Q2 and Qinyou8 were MYB and bHLH, respectively. It may be one of the important reasons for the different regulation modes of the two genotypes’ response to drought stress and re-watering. Nuclear factor Y (NF-Y) is composed of three distinct subunits (NF-YA, NF-YB, and NF-YC). We found that the *Arabidopsis thaliana* NFYA5 transcript is strongly induced by abscisic acid (ABA)-dependent manner under drought stress, and, the overexpressing of NFYA5 in *Arabidopsis thaliana* resisted drought stress by controlling stomatal aperture so as to reduce leaf water loss [115]. In this study, NFY accounted for the largest proportion of co-expressed TFs in the two genotypes, respectively. In summary, the two genotypes have different ways of responding to drought stress and re-watering, which is conducive to understanding the molecular regulatory mechanism in response to drought stress, and strengthening our understanding of drought regulatory network.

LncRNA HID1 (HIDDEN TREASURE 1) has been proved to be an important participant in seeding light morphogenesis by regulating PIF3 (phytochrome-interacting factor 3) expression [116]. In Chinese cabbage (*Brassica rapa ssp. chinensis*), some TFs were cis-regulated by the response of lncRNAs to heat stress [47]. Under water stress and during recovery, 189 TFs corresponded to 163 differentially expressed lncRNAs in *C. songorica*, and there was a bZIP gene predicted to be the target gene of an lncRNA (MSTRG.17203.1) [72]. These studies indicated that there was a regulatory relationship between lncRNAs and TFs. In total, 57 and 94 TFs related to 20 and 24 different families showed co-expression with lncRNAs in two genotypes, respectively. Though, the number of TFs and TF families co-expressed in Qinyou8 higher than Q2, but the occurrence pattern was comparable. The TFs related to the HSF, NF-YA, ERF, bHLH, MYB, GATA, and bZIP families were highly represented in Q2. Similarly, HSF, NF-YA, ERF, bHLH, MYB, WRKY, and bZIP TF families were more enriched in Qinyou8. Among specifically expressed TFs in Q2, a PAT1 gene (BnaC07g49170D) was predicted to be XLOC_096112 target gene and a TGA3 (BnaC05g17700D) was predicted to be XLOC_032712 target gene. In Qinyou8, a bHLH69 gene (BnaC01g07430D) was predicted to be a target gene for 10 lncRNAs. In our research, we also found that a bZIP gene (BnaA09g03330D) was predicted to be a target gene for 7 lncRNAs in Q2 and two bZIP genes (BnaA09g03330D and BnaA09g19470D) were predicted to be the target genes for 35 lncRNAs in Qinyou8. This result suggested that the regulation of lncRNAs might play crucial roles in response to drought stress. This would be the next step to explore.

### Other lncRNAs involved in drought stress and re-watering

Some other candidate functional and regulatory lncRNAs have been detected in response to drought stress and re-watering. We identified that XLOC_052298 and XLOC_094954 were down-regulated in the tolerant genotype and up-regulated in the susceptible genotype, XLOC_012868 was up-regulated in the tolerant genotype and down-regulated in the susceptible genotype. It was noticed that some mRNAs which were co-located with three lncRNAs, were mainly categorized into two categories, i.e. signal transport and defense/stress response.

Drought signals may be perceived by changes in membrane receptor activity. At this time, extracellular signals are converted into intracellular signals, which can lead to the production of second messengers such as Ca^2+^, sugars, ROS and IP_3_ delivery systems [117], triggering phosphorylation/dephosphorylation reactions and transmitting information, thereby activating specific transcription factors. After binding to the corresponding cis-acting elements, transcription factors regulate the expression of drought-stress-responsive genes [118]. Serine/threonine protein phosphatase is one of the major enzymes that catalyze the dephosphorylation of proteins [119]. A previous study has demonstrated that serine/threonine protein phosphatase is related to the regulation of anti-reverse signal transduction induced by abscisic acid in plants [120, 121]. As the core component of BR signaling, the BES1/BZR1 transcription factors are activated by the BR signal, bind to the E-box (CANNTG) or BRRE element (CGTGT/CG) of the growth and development-related genes promoter and regulate target gene expression [122–124]. BRs, an important plant hormone, improves drought resistance of plants by improving plant osmotic regulation and influencing the activities of antioxidative enzymes [125, 126]. Under drought stress, the accumulation of soluble sugars, such as trehalose, has the function of stabilizing the proteins and cell membranes, which is beneficial for the regulation of the balance between the osmotic pressure and the outside of the plant cells [127, 128]. Plants with reduced gibberellin (GA) activity, and therefore reduced transpiration, suffer less from leaf desiccation, thereby maintaining higher capabilities and recovery rates [129]. In this study, BnaC02g25020D, BnaC02g25150D and BnaC02g25200D, which co-locate with XLOC_052298, were associated with alpha-trehalose-phosphate synthase, peroxidase, and the BES1/BZR1 homolog protein, respectively. BnaC09g24140D, which co-locates with XLOC_094954, was associated with serine/threonine-protein phosphatase. BnaA03g47140D and BnaA03g47400D, which co-locate with XLOC_012868, were associated with superoxide dismutase, gibberellin oxidase, respectively. BnaA03g47370D and BnaA03g47380D, which co-locate with XLOC_012868, were associated with bHLH. Therefore, we believe that these lncRNAs may be related to drought stress and re-watering. However, our knowledge about the potential functions of these dysregulated lncRNAs in response to drought, remains limited. Thus, further investigation is of.

great importance.

## Conclusion

In this study, 5546 down-regulated and 6997 up-regulated mRNAs were detected in Q2, as compared to 7824 and 10,251 in Qinyou8, respectively; 369 down-regulated and 108 up-regulated lncRNAs were detected in Q2, compared with 449 and 257 in Qinyou8, respectively. In addition, the interaction networks between lncRNAs and mRNAs were constructed and the function of lncRNAs was then investigated based on the lncRNA-mRNA in-teraction networks. This study found that 4 lncRNAs were annotated significantly to the ABA signaling pathway within a KEGG pathway “plant hormone signal transduction”, in Q2, under drought stress and re-watering. Eight mRNAs, which co-locate with three lncRNAs, were mainly categorized into signal transport and defense/stress response under drought stress and re-watering. At the same time, the photosynthesis-associated genes were commonly up-regulated by drought stress and re-watering treatment in Qinyou8. In conclusion, the foregoing outcome indicates that drought stress and re-watering affects the expression of some lncRNAs, and the inter-regulation of lncRNAs and mRNAs may elicit response to drought stress and re-watering. While these findings provide newfound information regarding the potential role of lncRNAs in response to drought stress and re-watering, further research is required to elucidate the molecular mechanisms of significantly dysregulated lncRNAs. The co-expression network suggests that the inter-regulation of lncRNAs and mRNAs is involved in responses to drought stress and re-watering.

## Methods

### Plant materials, growth conditions, and treatments

Seeds of two contrasting rapeseed (*Brassica napus* L.) genotypes, Q2 (drought-tolerant) and Qinyou8 (drought-sensitive) were obtained from Oil Crops Research Institute (OCRI), Chinese Academy of Agricultural Sciences (CAAS), Wuhan, China. These two contrasting rapeseed genotypes were selected by analyzing the photosynthetic rate, chlorophyll content, carotenoid content, malondialdehyde content, and antioxidants activity of leaves under water stress [130, 131]. Under drought stress, Q2 had a relatively higher net photosynthetic rate, the relative water content (RWC), chlorophyll content, carotenoid content, and antioxidants activity in leaves than Qinyou8 [130–133].

The experiment was conducted in a greenhouse at 25 °C, with a photoperiod of 16 h of light and 8 h of darkness, in June 2017, and a humidity rate of 83%. The detailed preparation of the seeds and soils in pots were according to in Xiong et al. [132]. All pots were watered to 75% FC for 18 d (the three-leaf stage) with daily watering, before being subjected to drought stress. Experiment treatment conditions were as follows: (1) 18 days old plants were subjected to water deficit by leaving them un-watered for 8 days (set as drought stress, DS); (2) 18 days old plants were subjected to water deficit by leaving them un-watered for 7 days down to 35% FC [133] and then re-watered for 1 day to 75% FC (set as re-watering, RW). The experiment was carried out using a completely randomized design with three replications. After 8 d of treatment, the 3rd leaves were separately sampled from 5 individuals under each treatment from each replicate (12 samples in total) and quickly stored individually in liquid N_2_.

### Determination of physiological parameters

The uniform seedlings of each replicate under the WW, DS and RW treatments were used to measure fresh weight.

### RNA extraction, library construction, and Illumina sequencing

The process of RNA extraction and purity were according to Hu et al. [94]. A amount of 3 μg RNA per sample were used to generate cDNA libraries and sequenced. The qualified cDNA libraries were constructed by PCR enrichment and sequenced on a HiSeq X Ten with a sequencing read length of PE125. The 12 gene expression libraries were named DSQ2–1, DSQ2–2, DSQ2–3, RWQ2–1, RWQ2–2, RWQ2–3, DSQinyou8–1, DSQinyou8–2, DSQinyou8–3, RWQingyou8–1, RWQinyou8–2, and RWQinyu8–3. The library preparation and deep sequencing were performed by the Novogene Bioinformatics Technology Cooperation (Beijing, China). All the clean reads, obtained after the quality-control process, were deposited in the NCBI Sequence Read Archive with the ID PRJNA574049 for data analysis, as given in the following section.

### Mapping to the reference genome

Reference genome and gene model annotation files were downloaded from a genome website (http://brassicadb.org/brad/datasets/pub/Genomes/) directly. Index of the reference genome was built using bowtie v2.0.6 and paired-end clean reads were aligned to the reference genome using TopHat v2.0.9

### Quantification of gene expression level

Cuffdiff was used to calculate FPKMs (fragments per kilo-base of exon per million fragments mapped) of both lncRNAs and coding genes in each sample [134]. Values were calculated based on the length of the fragments and reads count mapped to this fragment. Gene FPKMs were computed by summing the FPKMs of the transcripts in each gene group.

### Differential expression analysis

Cuffdiff provides statistical routines for determining differential expression in digital transcript or gene expression data using a model based on the negative binomial distribution [134]. The expression strength of each gene was measured by the FPKM method [134] and calculated through averaging expression data of three replicates. The differentially expressed lncRNAs and mRNAs between samples were confirmed by Cufflinks software with the shreshold of q value ≤0.05 and a |log2(FPKM) ratio| ≥ 1. The calculation of *q* value was according to Trapnell et al. [134]. The treatments of RWQ2/DSQ2 and RWQinyou8/DSQinyou8 were performed.

### Construction of the lncRNA-mRNA co-expression network

LncRNA-mRNA co-expression networks were constructed to identify the interactions between protein-coding genes and lncRNAs according to the normalized signal intensities of the specific expression in genes and lncRNAs [135]. We constructed the lncRNA-mRNA co-expression network according to Wang et al. (2018) [136]. Firstly, the expression values of the differentially expressed lncRNAs and mRNAs were obtained. Secondly, the correlation between the differentially expressed lncRNAs and mRNAs was evaluated using the Pearson’s correlation coefficient (PCC) from matched mRNA and lncRNA expression profile data. The lncRNA-mRNA pairs with |PCC value ≥0.95| and *p* < 0.05 were selected as co-regulated lncRNA-mRNA pairs. Subsequently, the network was constructed, in which nodes were lncRNAs or mRNAs. In total, the lncRNA-mRNA co-expression networks were initially constructed based on co-expressed lncRNA-mRNA pairs in each comparison (RWQ2/DSQ2, RWQinyou8/DSQinyou8). Ultimately, to visually display the relationship between lncRNAs and target protein-coding RNAs, the interactive networks were constructed using Cytoscape software (3.7.1), (an open source software platform for visualizing complex networks available from http://cytoscape.org/).

### Function classification of the target genes of differentially expressed lncRNAs

Gene Ontology (GO) enrichment analysis of the target genes which co-expressed with differentially expressed lncRNAs were implemented using the GOseq R package, in which gene length bias was corrected. GO terms with corrected *p* value less than 0.05 were considered significantly enriched with differential expressed genes. We used KOBAS software to test the statistical enrichment of the target genes which co-expressed with differentially expressed lncRNAs in KEGG pathways. The most enriched KEGG was enlisted in order according to the corrected *p* value. A corrected *p* value < 0.05 was required for differences to be considered statistically significant.

### qRT-PCR analysis

After treated with RNase–free DNase, the RNA samples were used to generate cDNA by using the RevertAid First Strand cDNA Synthesis Kit (Fermentas, USA). Real-time PCR was performed on the ABI 700 platform with the SYBR Green PCR Master Mix system (Takara Co. Ltd., Japan). The 10 μl reaction volume in each well contained 0.5 ng cDNA, 2.5 μl of a mixture containing 1.2 μM each of the forward and reverse primers and 5 μl of master mix. The PCR amplification procedures were as: one iniative cycle of 30 s at 95 °C; followed by denaturation at 94 °C for 30 s, primer annealing at 60 °C for 30 s, and then extension at 72 °C for 1 min; finally, an extra extension at 72 °C for 10 min. The primer sequences for the randomly selected lncRNAs were shown in Additional file 9. Each PCR reaction were repeated three times independent and the expression strength of each lncRNA was set as their average value.

## Supplementary information

**Additional file 1.** RNA-seq data for 12 samples.

**Additional file 2.** RNA amount obtained from each treatment for RNA-seq analysis.

**Additional file 3.** Relative expression data measured by qRT-PCRs and RNA-seq.

**Additional file 4.** LncRNA and their putative target mRNA based on the co-expression results.

**Additional file 5.** The lncRNA-mRNA pairs reflected the opposite expression trend in two genotypes.

**Additional file 6.** Transcription factors corresponding to differentially expressed lncRNAs in two genotypes.

**Additional file 7.** Differentially expressed lncRNAs in response to drought stress and re-watering.

**Additional file 8.** The lncRNA-mRNA pairs in the lncRNA-mRNA co-expression network of plant hormone signal transduction.

**Additional file 9.** List of primers for quantitative real-time PCR.

## Data Availability

The datasets analyzed during the current study are available in the Sequence Read Archive (SRA) at NCBI (SRA accession: PRJNA574049) repository, https://www.ncbi.nlm.nih.gov/sra/PRJNA574049

## Abbreviations

A-ARR: Type-A response regulators
ABA: Abscisic acid
ABF: Abscisic binding factor
AHKs: Arabidopsis histidine kinases
AHPs: Arabidopsis histidine phosphotransfer proteins
AIB: ABA-inducible bHLH-type transcription factor
ARF: Auxin response factor
ARRs: Arabidopsis response regulators
Aux/IAA: Auxin / indole-3-acetic acid
B-ARR: Type-B response regulators
BES1/BZR1: bri1-Ethylmethane sulphonate suppressor 1 / brassinazole-resistant 1
bHLH: Basic helix-loop-helix
bZIP: Basic leucine zipper domain
CBF/DREB: C-repeat binding factor/dehydrate responsive element binding transcription factor
DRIR: Drought induced lncRNA
DS: Drought stress
ERF: Ethylene responsive element binding factors
FC: Field capacity
FPKM: Fragments per kilobase of transcript per million mapped reads
GA: Gibberellin acid
GH3: Gretchen hagen3
GID1: Gibberellin-insensitive dwarf 1
GO: Gene ontology
HID1: Hidden treasure 1
HSF: Heat-shock factor
IAA: Indole-3-acetic acid
IP3: Inositol 1, 4, 5-trisphosphate
KEGG: Kyoto encyclopedia of genes and genomes
LncRNAs: Long non-coding RNAs
MYB: Myeloblastosis
MYC: Myelocytomatosis
PCC: Pearson’s correlation coefficient
PIF3: Phytochrome-interacting factor 3
PP2C: Protein phosphatases type-2C
PYR/PYL/RCAR: Pyrabactin resistant / PYR-like / regulatory component of ABA
qRT-PCR: Quantitive Real-time PCR
RNA-seq: RNA sequencing
ROS: Reactive oxygen species
RW: Re-watering
RWC: Relative water content
SAUR: Small auxin up RNAs
SnRK2: Sucrose non-fermenting 1-related subfamily 2 kinases
TCS: Two-component signaling systems
TFs: Transcription factors
TIR1/AFBs: Transport inhibitor response 1 / Auxin signaling f-box proteins
WW: Well-watered

## References

[1] Wang W, Vinocur B, Altman A (2003). Plant responses to drought, salinity and extreme temperatures: towards genetic engineering for stress tolerance. Planta..

[2] Breshears DD, Cobb NS, Rich PM, Price KP, Allen CD, Balice RG (2005). Regional vegetation die-off in response to global-change-type drought. Proc Natl Acad Sci U S A.

[3] Coumou D, Robinson A (2013). Historic and future increase in the global land area affected by monthly heat extremes. Environ Res Lett.

[4] Ahuja I, de Vos RCH, Bones AM, Hall RD (2010). Plant molecular stress responses face climate change. Trends Plant Sci.

[5] Dai A (2013). Increasing drought under global warming in observations and models. Nat Clim Chang.

[6] Fang YJ, Xiong LZ (2015). General mechanisms of drought response and their application in drought resistance improvement in plants. Cell Mol Life Sci.

[7] Rabara RC, Tripathi P, Reese RN, Rushton DL, Alexander D, Timko MP (2015). Tobacco drought stress responses reveal new targets for solanaceae crop improvement. BMC Genomics.

[8] Fracasso A, Trindade LM, Amaducci S (2016). Drought stress tolerance strategies revealed by RNA-Seq in two sorghum genotypes with contrasting WUE. BMC Plant Biol.

[9] Xiong L, Schumaker KS, Zhu JK (2002). Cell signaling during cold, drought, and salt stress. Plant Cell.

[10] Huang GT, Ma SL, Bai LP, Zhang L, Ma H, Jia P (2012). Signal transduction during cold, salt, and drought stresses in plants. Mol Biol Rep.

[11] Vinocur B, Altman A (2005). Recent advances in engineering plant tolerance to abiotic stress: achievements and limitations. Curr Opin Biotechnol.

[12] Mittler R, Finka A, Goloubinoff P (2012). How do plants feel the heat?. Trends Biochem Sci.

[13] Farooq M, Wahid A, Kobayashi N, Fujita D, Basra S (2009). Plant drought stress: effects, mechanisms and management. Agron Sustain Dev.

[14] Mercer TR, Dinger ME, Mattick JS (2009). Long non-coding RNAs: insights into functions. Nat Rev Genet.

[15] Kowalczyk M, Higgs D, Gingeras T (2012). Molecular biology: RNA discrimination. Nature..

[16] Kung JTY, Colognori D, Lee JT (2013). Long noncoding RNAs: past, present, and future. Genetics..

[17] Sunkar R, Chinnusamy V, Zhu J, Zhu JK (2007). Small RNAs as big players in plant abiotic stress responses and nutrient deprivation. Trends Plant Sci.

[18] Ben Amor B, Wirth S, Merchan F, Laporte P, D’Aubenton-Carafa Y, Hirsch J (2009). Novel long non-protein coding RNAs involved in *Arabidopsis* differentiation and stress responses. Genome Res.

[19] Caley D, Pink R, Trujillano D, Carter D (2010). Long noncoding RNAs, chromatin, and development. ScientificWorldJournal..

[20] Ng JH, Ng HH (2010). LincRNAs join the pluripotency alliance. Nat Genet.

[21] Nagano T, Fraser P (2011). No-nonsense functions for long noncoding RNAs. Cell..

[22] Ding J, Lu Q, Ouyang Y, Mao H, Zhang P, Yao J (2012). A long noncoding RNA regulates photoperiod-sensitive male sterility, an essential component of hybrid rice. Proc Natl Acad Sci U S A.

[23] Kim ED, Sung S (2012). Long noncoding RNA: unveiling hidden layer of gene regulatory networks. Trends Plant Sci.

[24] Moran V, Perera R, Khalil A (2012). Emerging functional and mechanistic paradigms of mammalian long non-coding RNAs. Nucleic Acids Res.

[25] Kornienko AE, Guenzl PM, Barlow DP, Pauler FM (2013). Gene regulation by the act of long non-coding RNA transcription. BMC Biol.

[26] Di C, Yuan JP, Wu Y, Li JR, Lin HX, Hu L (2014). Characterization of stress-responsive lncRNAs in *Arabidopsis thaliana* by integrating expression, epigenetic and structural features. Plant J.

[27] Zhu QH, Stephen S, Taylor J, Helliwell CA, Wang MB (2014). Long noncoding RNAs responsive to Fusarium oxysporum infection in *Arabidopsis thaliana*. New Phytol.

[28] Wu J, Okada T, Fukushima T, Tsudzuki T, Sugiura M, Yukawa Y (2012). A novel hypoxic stress-responsive long non-coding RNA transcribed by RNA polymerase III in *Arabidopsis*. RNA Biol.

[29] Liu J, Jung C, Xu J, Wang H, Deng S, Bernad L (2012). Genome-wide analysis uncovers regulation of long intergenic noncoding RNAs in *Arabidopsis*. Plant Cell.

[30] Zhao X, Liu X, Guo C, Gu J, Xiao K (2013). Identification and characterization of microRNAs from wheat (*Triticum aestivum* L.) under phosphorus deprivation. J Plant Biochem Biotechnol.

[31] Zhang W, Han ZX, Guo QL, Liu Y, Zheng YX, Wu FL (2014). Identification of maize long non-coding RNAs responsive to drought stress. PLoS One.

[32] Li L, Eichten SR, Shimizu R, Petsch K, Yeh CT, Wu W (2014). Genome-wide discovery and characterization of maize long non-coding RNAs. Genome Biol.

[33] Lv Y, Liang ZK, Ge M, Qi WC, Zhang TF, Lin F (2016). Genome-wide identification and functional prediction of nitrogen-responsive intergenic and intronic long non-coding RNAs in maize (*Zea mays* L.). BMC Genomics.

[34] Zhang YC, Liao JY, Li ZY, Yu Y, Zhang JP, Li QF (2014). Genome-wide screening and functional analysis identify a large number of long noncoding RNAs involved in the sexual reproduction of rice. Genome Biol.

[35] Xin M, Wang Y, Yao Y, Song N, Hu Z, Qin D (2011). Identification and characterization of wheat long non-protein coding RNAs responsive to powdery mildew infection and heat stress by using microarray analysis and SBS sequencing. BMC Plant Biol.

[36] Zhang YC, Chen YQ (2013). Long noncoding RNAs: new regulators in plant development. Biochem Biophys Res Commun.

[37] Shuai P, Liang D, Tang S, Zhang Z, Ye CY, Su Y (2014). Genome-wide identification and functional prediction of novel and drought-responsive lincRNAs in *Populus trichocarpa*. J Exp Bot.

[38] Chung PJ, Jung H, Jeong DH, Ha S, Do CY, Kim J (2016). Transcriptome profiling of drought responsive noncoding RNAs and their target genes in rice. BMC Genomics.

[39] Lu XK, Chen XQ, Mu M, Wang JJ, Wang XG, Wang DL (2016). Genome-wide analysis of long noncoding RNAs and their responses to drought stress in cotton (*Gossypium hirsutum* L.). PLoS One.

[40] Qi X, Xie SJ, Liu YW, Yi F, Yu JJ (2013). Genome-wide annotation of genes and noncoding RNAs of foxtail millet in response to simulated drought stress by deep sequencing. Plant Mol Biol.

[41] Qin T, Zhao HY, Cui P, Albesher N, Xiong LM (2017). A nucleus-localized long non-coding RNA enhances drought and salt stress tolerance. Plant Physiol.

[42] Li SX, Yu X, Lei N, Cheng ZH, Zhao PJ, He YK (2017). Genome-wide identification and functional prediction of cold and/or drought-responsive lncRNAs in cassava. Sci Rep.

[43] Ahmed Waqas, Xia Yanshi, Li Ronghua, Bai Guihua, Siddique Kadambot H.M., Guo Peiguo (2020). Non-coding RNAs: Functional roles in the regulation of stress response in Brassica crops. Genomics.

[44] Song XM, Liu GF, Huang ZN, Duan WK, Tan HW (2016). Temperature expression patterns of genes and their coexpression with LncRNAs revealed by RNA-Seq in non-heading Chinese cabbage. BMC Genomics.

[45] Zhang JF, Wei LJ, Jiang J, Mason AS, Li HJ (2018). Genome-wide identification, putative functionality and interactions between lncRNAs and miRNAs in *Brassica* species. Sci Rep.

[46] Joshi RK, Megha S, Basu U, Rahman MH, Kav NNV (2016). Genome wide identification and functional prediction of long non-coding RNAs responsive to *Sclerotinia sclerotiorum* infection in *Brassica napus*. PLoS One.

[47] Wang AH, Hu JH, Gao CB, Chen GL, Wang BC, Lin CF (2019). Genome-wide analysis of long non-coding RNAs unveils the regulatory roles in the heat tolerance of Chinese cabbage (*Brassica rapa ssp. chinensis*). Sci Rep.

[48] Shea DJ, Nishida N, Takada S, Itabashi E, Takahashi S, Akter A (2019). Long noncoding RNAs in *Brassica rapa* L following vernalization. Sci Rep.

[49] Summanwar A, Basu U, Rahman H, Kav N (2019). Identification of lncRNAs responsive to infection by plasmodiophora brassicae in clubroot-susceptible and -resistant *Brassica napus* lines carrying resistance introgressed from rutabaga. Mol Plant-Microbe Interact.

[50] Bhatia G, Singh A, Verma D, Sharma S, Singh K (2020). Genome-wide investigation of regulatory roles of lncRNAs in response to heat and drought stress in *Brassica juncea* (Indian mustard). Environ Exp Bot.

[51] Miller CN, Harper AL, Trick M, Wellner N, Werner P, Waldron KW (2018). Dissecting the complex regulation of lodging resistance in *Brassica napus*. Mol Breed.

[52] Khalili M, Aboughadareh AP, Naghavi MR, Javad S (2012). Response of spring canola (*Brassica napus* L.) genotypes to water deficit stress. Int J Agric Crop Sci.

[53] Müller T, Lentzsch P, Müller MEH (2012). Carbohydrate dynamics in leaves of rapeseed (*Brassica napus*) under drought. J Agron Crop Sci.

[54] Zhang J, Mason AS, Wu J, Liu S, Zhang XC, Luo T (2015). Identification of putative candidate genes for water stress tolerance in canola (*Brassica napus*). Front Plant Sci.

[55] Liao Q, Liu CN, Yuan XY, Kang SL, Miao R, Xiao H (2011). Large-scale prediction of long non-coding RNA functions in a coding-non-coding gene co-expression network. Nucleic Acids Res.

[56] Wilusz JE, Sunwoo H, Spector DL (2009). Long noncoding RNAs: functional surprises from the RNA world. Genes Dev.

[57] Xu J, Zhang F, Gao C, Ma XF, Peng XL, Kong DX (2017). Microarray analysis of lncRNA and mRNA expression profiles in patients with neuromyelitis optica. Mol Neurobiol.

[58] Wang TZ, Liu M, Zhao MG, Chen RJ, Zhang WH (2015). Identification and characterization of long non-coding RNAs involved in osmotic and salt stress in *Medicago truncatula* using genome-wide high-throughput sequencing. BMC Plant Biol.

[59] Faghihi MA, Modarresi F, Khalil AM, Wood DE, Sahagan BG, Morgan TE (2008). Expression of a noncoding RNA is elevated in Alzheimer’s disease and drives rapid feed-forward regulation of β-secretase. Nat Med.

[60] Camblong J, Iglesias N, Fickentscher C, Dieppois G, Stutz F (2007). Antisense RNA stabilization induces transcriptional gene silencing via histone deacetylation in *S. cerevisiae*. Cell..

[61] Houseley J, Rubbi L, Grunstein M, Tollervey D, Vogelauer M (2008). A ncRNA modulates histone modification and mRNA induction in the yeast GAL gene cluster. Mol Cell.

[62] Hung T, Wang YL, Lin MF, Koegel AK, Kotake Y, Grant GD (2011). Extensive and coordinated transcription of noncoding RNAs within cell-cycle promoters. Nat Genet.

[63] Tripathi V, Ellis JD, Shen Z, Song DY, Pan Q, Watt AT (2010). The nuclear-retained noncoding RNA MALAT1 regulates alternative splicing by modulating SR splicing factor phosphorylation. Mol Cell.

[64] Gong CG, Maquat LE (2011). LncRNAs transactivate STAU1-mediated mRNA decay by duplexing with 3′ UTRs via Alu elements. Nature..

[65] Mizoi J, Shinozaki K, Yamaguchi-Shinozaki K (2011). AP2/ERF family transcription factors in plant abiotic stress responses. Biochim Biophys Acta.

[66] Muthusamy M, Uma S, Suthanthiram B, Saraswathi M (2015). Genome-wide screening for novel, drought stress-responsive long non-coding RNAs in drought-stressed leaf transcriptome of drought-tolerant and -susceptible banana (*Musa* spp) cultivars using Illumina high-throughput sequencing. Plant Biotechnol Rep.

[67] Marquardt S, Raitskin O, Wu Z, Liu FQ, Sun QW, Dean C (2014). Functional consequences of splicing of the antisense transcript *COOLAIR* on *FLC* transcription. Mol Cell.

[68] Bardou F, Ariel F, Simpson CG, Romero-Barrios N, Laporte P, Balzergue S (2014). Long noncoding RNA modulates alternative splicing regulators in *Arabidopsis*. Dev Cell.

[69] Chen L, Shi SL, Jiang NF, Khanzada H, Wassan GM, Zhu CL (2018). Genome-wide analysis of long non-coding RNAs affecting roots development at an early stage in the rice response to cadmium stress. BMC Genomics.

[70] Huanca-Mamani W, Arias-Carrasco R, Cárdenas-Ninasivincha S, Rojas-Herrera M, Sepúlveda-Hermosilla G, Caris-Maldonado JC (2018). Long non-coding RNAs responsive to salt and boron stress in the hyper-arid lluteño maize from Atacama desert. Genes (Basel).

[71] Chen JH, Quan MY, Zhang DQ (2014). Genome-wide identification of novel long non-coding RNAs in *Populus tomentosa* tension wood, opposite wood and normal wood xylem by RNA-seq. Planta..

[72] Yan Q, Wu F, Yan ZZ, Li J, Ma TT, Zhang YF (2019). Differential co-expression networks of long non-coding RNAs and mRNAs in *Cleistogenes songorica* under water stress and during recovery. BMC Plant Biol.

[73] Wang JX, Lin J, Kan JL, Wang H, Li XG, Yang QS (2018). Genome-wide identification and functional prediction of novel drought-responsive lncRNAs in *Pyrus betulifolia*. Genes (Basel).

[74] Zhang C, Tang GJ, Peng X, Sun FL, Liu SD, Xi YJ (2018). Long non-coding RNAs of switchgrass (*Panicum virgatum* L.) in multiple dehydration stresses. BMC Plant Biol.

[75] Kilian J, Whitehead D, Horak J, Wanke D, Weinl S, Batistic O (2007). The AtGenExpress global stress expression data set: protocols, evaluation and model data analysis of UV-B light, drought and cold stress responses. Plant J.

[76] Yuan S, Liu WJ, Zhang NH, Wang MB, Liang HG, Lin HH (2005). Effects of water stress on major PSII gene expression and protein metabolism in barley leaves. Physiol Plant.

[77] Shinozaki K, Yamaguchi-Shinozaki K (1997). Gene expression and signal transduction in water-stress response. Plant Physiol.

[78] Schachtman DP, Goodger JQD (2008). Chemical root to shoot signaling under drought. Trends Plant Sci.

[79] Teale WD, Paponov IA, Palme K (2006). Auxin in action: signalling, transport and the control of plant growth and development. Nat Rev Mol Cell Biol.

[80] Ljung K (2013). Auxin metabolism and homeostasis during plant development. Development..

[81] Abel S, Oeller PW, Theologis A (1994). Early auxin-induced genes encode short-lived nuclear proteins. Proc Natl Acad Sci U S A.

[82] Tiwari SB, Wang XJ, Hagen G, Guilfoyle TJ (2001). AUX/IAA proteins are active repressors, and their stability and activity are modulated by auxin. Plant Cell.

[83] Staswick PE, Serban B, Rowe M, Tiryaki I, Maldonado MT, Maldonado MC, Suza W (2005). Characterization of an Arabidopsis enzyme family that conjugates amino acids to indole-3-acetic acid. Plant Cell.

[84] Hwang I, Sheen J, Müller B (2012). Cytokinin signaling networks. Annu Rev Plant Biol.

[85] Müller B (2011). Generic signal-specific responses: cytokinin and context-dependent cellular responses. J Exp Bot.

[86] Pimenta Lange MJ, Lange T (2006). Gibberellin biosynthesis and the regulation of plant development. Plant Biol (Stuttg).

[87] Zentella R, Zhang ZL, Park M, Thomas SG, Endo A, Murase K (2007). Global analysis of DELLA direct targets in early gibberellin signaling in *Arabidopsis*. Plant Cell.

[88] Fleet CM, Sun TP (2005). A DELLAcate balance: the role of gibberellin in plant morphogenesis. Curr Opin Plant Biol.

[89] Zhu JK (2002). Salt and drought stress signal transduction in plants. Annu Rev Plant Biol.

[90] Zhang X, Takemiya A, Kinoshita T, Shimazaki K (2007). Nitric oxide inhibits blue light-specific stomatal opening via abscisic acid signaling pathways in Vicia guard cells. Plant Cell Physiol..

[91] Cutler SR, Rodriguez PL, Finkelstein RR, Abrams SR (2010). Abscisic acid: emergence of a core signaling network. Annu Rev Plant Biol.

[92] Ben-Ari G (2012). The ABA signal transduction mechanism in commercial crops: learning from Arabidopsis. Plant Cell Rep.

[93] Wang P, Yang CL, Chen H, Song CP, Zhang X, Wang DJ (2017). Transcriptomic basis for drought-resistance in *Brassica napus* L. Sci Rep.

[94] Hu HH, Xiong LZ (2014). Genetic engineering and breeding of drought-resistant crops. Annu Rev Plant Biol.

[95] Shinozaki K, Yamaguchi-Shinozaki K (2007). Gene networks involved in drought stress response and tolerance. J Exp Bot.

[96] Zhang XJ, Liu XY, Zhang DF, Tang HJ, Sun BC (2017). Genome-wide identification of gene expression in contrasting maize inbred lines under field drought conditions reveals the significance of transcription factors in drought tolerance. PLoS One.

[97] Shin DJ, Moon SJ, Han S, Kim BG, Park SR, Lee SK (2011). Expression of *StMYB1R-1*, a novel potato single MYB-like domain transcription factor, increases drought tolerance. Plant Physiol.

[98] Bi HH, Luang S, Li Y, Bazanova N, Morran S, Song ZH (2016). Identification and characterization of wheat drought-responsive MYB transcription factors involved in the regulation of cuticle biosynthesis. J Exp Bot.

[99] Castilhos G, Lazzarotto F, Spagnolo-Fonini L, Bodanese-Zanettini MH, Margis-Pinheiro M (2014). Possible roles of basic helix-loop-helix transcription factors in adaptation to drought. Plant Sci.

[100] Dong Y, Wang CP, Han X, Tang S, Liu S, Xia XL, Yin WL (2014). A novel bHLH transcription factor *PebHLH35* from *Populus euphratica* confers drought tolerance through regulating stomatal development, photosynthesis and growth in *Arabidopsis*. Biochem Biophys Res Commun.

[101] Okay S, Derelli E, Unver T (2014). Transcriptome-wide identification of bread wheat WRKY transcription factors in response to drought stress. Mol Gen Genomics.

[102] Trujillo LE, Sotolongo M, Menéndez C, Ochogavía ME, Coll Y, Hernández I (2008). SodERF3, a novel sugarcane ethylene responsive factor (ERF), enhances salt and drought tolerance when overexpressed in tobacco plants. Plant Cell Physiol.

[103] Quach TN, Nguyen HTM, Valliyodan B, Joshi T, Xu D, Nguyen HT (2015). Genome-wide expression analysis of soybean *NF-Y* genes reveals potential function in development and drought response. Mol Gen Genomics.

[104] Gupta P, Nutan KK, Singla-Pareek SL, Pareek A (2017). Abiotic stresses cause differential regulation of alternative splice forms of GATA transcription factor in rice. Front Plant Sci.

[105] Gao Y, Jiang W, Dai Y, Xiao N, Zhang CQ, Li H (2015). A maize phytochrome-interacting factor 3 improves drought and salt stress tolerance in rice. Plant Mol Biol.

[106] Kudo M, Kidokoro S, Yoshida T, Mizoi J, Todaka D, Fernie AR (2017). Double overexpression of DREB and PIF transcription factors improves drought stress tolerance and cell elongation in transgenic plants. Plant Biotechnol J.

[107] Liu CQ, Zhang XK, Zhang K, An H, Hu KN, Wen J (2015). Comparative analysis of the Brassica napus root and leaf transcript profiling in response to drought stress. Int J Mol Sci.

[108] Guo M, Liu JH, Ma X, Luo DX, Gong ZH, Lu MH (2016). The plant heat stress transcription factors (HSFs): structure, regulation, and function in response to abiotic stresses. Front Plant Sci.

[109] Kang C, Zhai H, He SZ, Zhao N, Liu QC (2019). A novel sweetpotato bZIP transcription factor gene, *IbbZIP1*, is involved in salt and drought tolerance in transgenic *Arabidopsis*. Plant Cell Rep.

[110] Cui FQ, Brosché M, Lehtonen MT, Amiryousefi A, Xu EJ, Punkkinen M (2016). Dissecting abscisic acid signaling pathways involved in cuticle formation. Mol Plant.

[111] Liang YK, Dubos C, Dodd IC, Holroyd GH, Hetherington AM, Campbell MM (2005). *At*MYB61, an R2R3-MYB transcription factor controlling stomatal aperture in *Arabidopsis thaliana*. Curr Biol.

[112] Jung C, Seo JS, Han SW, Koo YJ, Kim CH, Song SI (2008). Overexpression of *AtMYB44* enhances stomatal closure to confer abiotic stress tolerance in transgenic Arabidopsis. Plant Physiol.

[113] Li HM, Sun JQ, Xu YX, Jiang HL, Wu XY, Li CY (2007). The bHLH-type transcription factor AtAIB positively regulates ABA response in *Arabidopsis*. Plant Mol Biol.

[114] Seo JS, Joo J, Kim MJ, Kim YK, Nahm BH, Song SI (2011). OsbHLH148, a basic helix-loop-helix protein, interacts with OsJAZ proteins in a jasmonate signaling pathway leading to drought tolerance in rice. Plant J.

[115] Li WX, Oono Y, Zhu JH, He XJ, Wu JM, Iida K (2008). The *Arabidopsis* NFYA5 transcription factor is regulated transcriptionally and posttranscriptionally to promote drought resistance. Plant Cell.

[116] Wang YQ, Fan XD, Lin F, He GM, Terzaghi W, Zhu DM, Deng XW (2014). *Arabidopsis* noncoding RNA mediates control of photomorphogenesis by red light. Proc Natl Acad Sci U S A.

[117] Osakabe Y, Arinaga N, Umezawa T, Katsura S, Nagamachi K, Tanaka H (2013). Osmotic stress responses and plant growth controlled by potassium transporters in *Arabidopsis*. Plant Cell.

[118] Chinnusamy V, Schumaker K, Zhu JK (2004). Molecular genetic perspectives on cross-talk and specificity in abiotic stress signalling in plants. J Exp Bot.

[119] Broz AK, Thelen JJ, Muszynski MG, Miernyk JA, Randall DD (2001). ZMPP2, a novel type-2C protein phosphatase from maize. J Exp Bot.

[120] Vranová E, Langebartels C, Van Montagu M, Inzé D, Van Camp W (2000). Oxidative stress, heat shock and drought differentially affect expression of a tobacco protein phosphatase 2C. J Exp Bot.

[121] Meskiene I, Baudouin E, Schweighofer A, Liwosz A, Jonak C, Rodriguez PL (2003). Stress-induced protein phosphatase 2C is a negative regulator of a mitogen-activated protein kinase. J Biol Chem.

[122] Oh E, Zhu JY, Wang ZY (2012). Interaction between BZR1 and PIF4 integrates brassinosteroid and environmental responses. Nat Cell Biol.

[123] Qiao SL, Sun SY, Wang LL, Wu ZH, Li CX, Li XM (2017). The RLA1/SMOS1 transcription factor functions with OsBZR1 to regulate brassinosteroid signaling and rice architecture. Plant Cell.

[124] Ye HX, Li L, Guo HQ, Yin YH (2012). MYBL2 is a substrate of GSK3-like kinase BIN2 and acts as a corepressor of BES1 in brassinosteroid signaling pathway in *Arabidopsis*. Proc Natl Acad Sci U S A.

[125] Yuan GF, Jia CG, Li Z, Sun B, Zhang LP, Liu N, Wang QM (2010). Effect of brassinosteroids on drought resistance and abscisic acid concentration in tomato under water stress. Sci Hortic (Amsterdam).

[126] Wang XX, Gao YG, Wang QJ, Chen M, Ye XL, Li DM (2019). 2, 4-Epibrassinolide-alleviated drought stress damage influences antioxidant enzymes and autophagy changes in peach (*Prunus persicae* L.) leaves. Plant Physiol Biochem.

[127] Goddijn OJM, van Dun K (1999). Trehalose metabolism in plants. Trends Plant Sci.

[128] Garg AK, Kim JK, Owens TG, Ranwala AP, Choi YD, Kochian LV, Wu RJ (2002). Trehalose accumulation in rice plants confers high tolerance levels to different abiotic stresses. Proc Natl Acad Sci U S A.

[129] Nir I, Moshelion M, Weiss D (2014). The Arabidopsis GIBBERELLIN METHYL TRANSFERASE 1 suppresses gibberellin activity, reduces whole-plant transpiration and promotes drought tolerance in transgenic tomato. Plant Cell Environ.

[130] Xiao QS. Drought-related gene expression analysis during drought stress in Rapeseed (*Brassica napus* L.) Master’s Thesis, Oil Crops Research Institute Chinese Academy of Agricultural Sciences, Wuhan, China, 2011.

[131] Li Z. Evaluation of drought tolerance in varieties of rapeseed (*Brassica napus* L.) and role ofexogenous GA3. Master’s Thesis, Oil Crops Research Institute Chinese Academy of Agricultural Sciences, Wuhan, China, 2010.

[132] Xiong JL, Dai LL, Ma N, Zhang CL (2018). Transcriptome and physiological analyses reveal that AM1 as an ABA-mimicking ligand improves drought resistance in *Brassica napus*. Plant Growth Regul.

[133] Naeem MS, Dai LL, Ahmad F, Ahmad A, Li J, Zhang CL (2016). AM1 is a potential ABA substitute for drought tolerance as revealed by physiological and ultra-structural responses of oilseed rape. Acta Physiol Plant.

[134] Trapnell C, Williams BA, Pertea G, Mortazavi A, Kwan G, van Baren MJ (2010). Transcript assembly and quantification by RNA-Seq reveals unannotated transcripts and isoform switching during cell differentiation. Nat Biotechnol.

[135] Pujana MA, Han JDJ, Starita LM, Stevens KN, Tewari M, Ahn JS, Rennert G (2007). Network modeling links breast cancer susceptibility and centrosome dysfunction. Nat Genet.

[136] Wang RH, Zou J, Meng JL, Wang JB (2018). Integrative analysis of genome-wide lncRNA and mRNA expression in newly synthesized *Brassica* hexaploids. Ecol Evol.

